# Recent Advances in Injectable Hydrogels for Biomedical and Aesthetic Applications: Focus on Rheological Characteristics

**DOI:** 10.3390/gels12010011

**Published:** 2025-12-23

**Authors:** Hyerin Lee, Yujin Jeong, Nayeon Lee, Inhye Lee, Jin Hyun Lee

**Affiliations:** 1 Department of Biowellness Convergence, Konkuk University, Chungju 27478, Republic of Korea; 2 School of Bio-Convergence Science, College of Biomedical & Health Science, Konkuk University, Chungju 27478, Republic of Korea

**Keywords:** injectable hydrogels, rheology, viscoelasticity, gelation, biomedical and aesthetic applications

## Abstract

Injectable hydrogels (IHs) have gained considerable interest in biomedical and aesthetic applications due to their minimally invasive delivery, selective localization, and sustained release of bioactive agents. They exhibit flowability during administration and undergo in situ gelation under physiological conditions. These behaviors are influenced by their tunable structural, physical, mechanical, and viscoelastic properties, modulating performance. Rheological parameters, including viscosity (*η*), storage modulus (*G*′), loss modulus (*G*″), and yield stress (*τ_y_*) of IHs with time (*t*), shear rate ($\overset{\cdot }{\gamma }$), and frequency (*f*), explaining their shear thinning, thixotropy, viscoelasticity, and gelatin kinetics, serve as key quantitative indicators of their injectability, self-healing capability, and structural and mechanical stability. The rheological characteristics reflect molecular interactions and crosslinking mechanisms within IH networks, thereby linking formulation to provide overall performance, including injectability, biodegradability, and controlled release. This review summarizes recent advances in IHs for diverse applications, with a primary focus on their rheological properties. It also briefly addresses their composition, intermolecular interactions, and correlated function and performance. The applications discussed include hemostatic and wound dressings, tissue engineering and regenerative medicine scaffolds, drug delivery systems, reconstructive and aesthetic materials, and functional bioinks for 3D printing. Overall, this review demonstrates that rheological characterization provides an essential framework for the rational engineering of next-generation IH systems.

## 1. Introduction

Injectable hydrogels (IHs), emerging as next-generation biomaterials, consist of hydrophilic polymer networks with high water-absorption and -retention capability and remain in a liquid state during administration through clinically relevant delivery channels under an acceptable injection force, subsequently undergoing gelation under physiological conditions to form stable three-dimensional networks [1,2,3]. This sol-gel transition enables in situ gelation, providing localized and minimally invasive delivery of active ingredients and achieving sustained effects at the target site [4,5]. Beyond their traditional biomedical and pharmaceutical uses, such as wound healing, tissue repair, and drug delivery, the IHs have also been extended to aesthetic applications, where precision, adaptability, and biocompatibility are also essential [6,7,8,9]. Continuous advancements in crosslinking strategies and formulation design, as well as in polymer chemistry, physics, and rheology, enable the expansion of the functionality of these systems, leading to a new generation of multifunctional injectable materials [10].

The formation of IHs is primarily governed by the crosslinking mechanism, the intermolecular interactions within the hydrogel matrix, and environmental conditions. Depending on the formulation strategy, the sol-gel transition occurs through physical crosslinking or chemical crosslinking, and often in response to environmental stimuli such as temperature, pH, light, or ionic strength [11,12]. Physically crosslinked IHs are stabilized through non-covalent interactions, including electrostatic and hydrogen bonding, hydrophobic associations, π–π stacking, and van der Waals forces, which provide reversibility, self-healing capability, and responsiveness to external environments [13,14,15,16]. In contrast, chemically crosslinked IHs are formed by covalent bonds through in situ reactions, such as Diels–Alder reaction, Michael addition, Schiff base condensation, and enzyme- or photo-mediated polymerization, yielding more durable, mechanically stable, and robust networks [13,16,17,18]. Moreover, hybrid IH systems that combine both physical and chemical crosslinking mechanisms have emerged to achieve a dynamic balance between adaptability and structural stability. Such interplay between reversible and permanent interactions governs not only gelation kinetics and viscoelasticity but also injectability, post-administration performance, and degradation kinetics [15,19,20]. By tailoring composition, crosslinking mechanism, and environmental responsiveness, IHs can be engineered with tunable structure, degradability, viscoelasticity, and mechanical and biological responses [21,22]. These design strategies allow IHs to maintain fluidity during injection while achieving network integrity at the administered site [23]. This feature is particularly beneficial in applications such as shape-conforming implants [24], localized drug delivery [25], and regenerative therapies [26].

**Figure 1:**
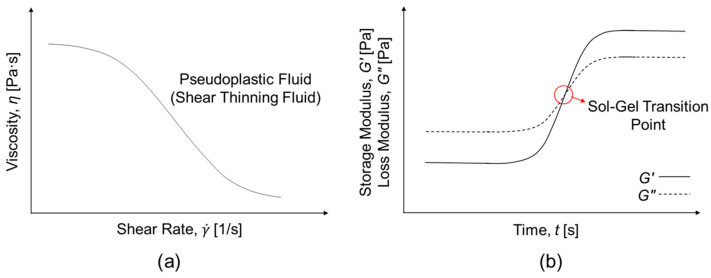
Representative rheological characteristics of injectable hydrogels (IHs). (**a**) Flow behavior as a function of shear rate for IHs (pseudoplastic fluid/shear thinning fluid); (**b**) Time-dependent gelation behavior, where the sol-gel transition point is identified at the crossover of *G*′ and *G*″.

Rheological behavior, among various material characteristics, profoundly influences performance and provides direct insight into how the microstructure of materials governs their macroscopic functionality [27,28]. The study of viscoelastic responses under deformation elucidates how readily a hydrogel precursor can be injected under an applied stress, how rapidly it forms or recovers its network structure once placed, and how effectively it resists external forces once gelation occurs [15]. The rheological characteristics of IHs are represented by key rheological parameters, such as viscosity (*η*), storage modulus (*G*′), loss modulus (*G*″), and yield stress (*τ*_y_), which depend on strain (*γ*), shear strain rate ($\overset{\cdot }{\gamma }$), frequency (*f*), and temperature (*T*). In conjunction with flow behaviors, including shear-thinning and recovery behaviors, these parameters collectively determine the injectability, viscoelasticity, mechanical strength, structural integrity, and functional reliability of IHs [29,30]. Accordingly, rheological behavior is a fundamental determinant of performance, serving as a bridge between molecular-level interaction-derived microstructure and macroscopic function, providing a quantitative framework for optimizing the formulation design and functional performance of IHs [27,31]. Figure 1 shows the representative rheological characteristics of IHs. Flow tests of IHs, evaluating their fluidity and injectability, provide information on their shear-thinning and thixotropic behavior. Time-sweep analysis monitors the gelation behavior of IHs, with the sol-gel transition identified at the crossover between *G*′ and *G*″. Besides, amplitude and frequency sweeps are performed to characterize the linear viscoelastic (LVE) region, structural stability, and the viscoelastic behavior of IHs before and after gelation. Moreover, self-healing and structural recovery behavior are monitored by creep-recovery or step-strain tests [30,31].

In addition to these conventional rheological properties, the injectability of IHs can be more quantitatively assessed using several indicators that influence syringe-type device-based extrusion behavior. These indicators include the critical injection force (*F_c_*) required to initiate extrusion through a delivery conduit, the shear thinning index (*n*) that reflects the reduced *η* under elevated shear conditions, and the sufficiently high $\overset{\cdot }{\gamma }$ generated by *F_c_* that lowers *η* enables smooth flow of IHs through needles, cannulas, and catheters, while maintaining appropriate post-injection $\overset{\cdot }{\gamma }$ and *η* to prevent tissue damage or necrosis. Moreover, the yield stress threshold that allows uninterrupted flow is essential for clog-free administration. Collectively, these indicators establish a generalized rheological framework for evaluating injectability across various delivery systems, such as clinical devices and administration routes.

This review presents an overview of recent studies on IHs for diverse biomedical and aesthetic applications, mainly focusing on their rheological properties. It also briefly addresses their composition, intermolecular interactions, and related function and performance. The diverse applications include hemostatic and wound dressings, tissue engineering and regenerative medicine scaffolds, drug delivery systems, reconstructive and aesthetic materials, and functional bioinks for 3D printing. By integrating viscoelastic characteristics with other features of IHs, this review aims to establish a conceptual framework that connects rheological parameters to practical function and performance. The summary of recent injectable hydrogels (IHs), their crosslinking mechanisms and associated intermolecular interactions, key features, and rheological properties across diverse applications is provided in Table 1, placed after the Conclusion section to enable collective examination of all IH systems. Moreover, quantitative rheological properties of natural and synthetic IHs, rheological and mechanical properties induced by different crosslinking mechanisms, and quantitative rheological and characteristic criteria for major IH applications are listed in Table 2, Table 3 and Table 4, also positioned after the Conclusion section and Table 1 for integrated comparison. Furthermore, current challenges and future perspectives are also discussed. Ultimately, this review offers a perspective that not only summarizes current knowledge but also identifies future directions for developing IH systems optimized for clinical reliability and personalized biomedical and aesthetic applications.

## 2. Recent Advances in IHs and Their Rheological Characteristics Across Diverse Biomedical and Aesthetic Applications

**Figure 2:**
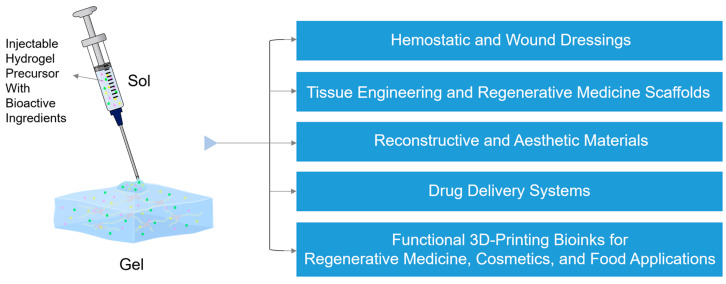
Diverse biomedical and aesthetic applications of IHs, with bioactive ingredients represented by the green dots, yellow five-pointed stars, and pink triangles: hemostatic and wound dressings, tissue engineering and regenerative medicine scaffolds, drug delivery systems, reconstructive and aesthetic materials, and functional 3D-printing bioinks for regenerative medicine, cosmetics, and food applications.

The rheological behavior of IHs varies considerably across intended applications, as each field requires a specific balance among injection flowability, gelation rate, and mechanical stability after administration [22,32,33]. In both biomedical and aesthetic applications, these parameters determine not only injectability, but also long-term functional stability once administered [34,35,36,37]. In this section, the biomedical and aesthetic applications of IHs are classified, according to their rheological functionality and performance requirements into six categories as follows: (1) hemostatic and wound-healing dressings, (2) tissue engineering and regenerative medicine scaffolds, (3) controlled-drug delivery systems, (4) reconstructive materials, (5) aesthetic materials, and (6) functional 3D-printing bioinks for regenerative medicine, cosmetics, and food applications (Figure 2). The recent advances in IHs used in these six applications are reviewed, mainly with regard to their rheological properties.

### 2.1. Hemostatic and Wound Dressings

**Figure 3:**
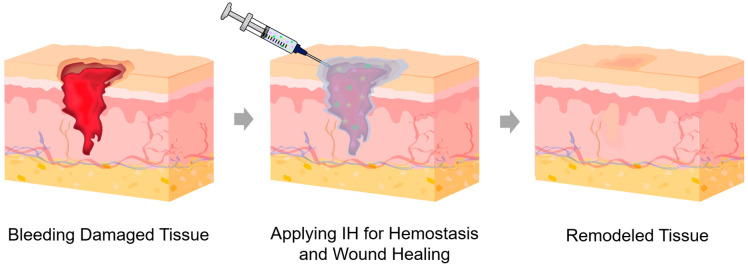
Schematic illustration of the hemostasis and wound-healing process using an IH: initial bleeding damaged tissue; application of an IH with bioactive ingredients represented by the green dots, yellow five-pointed stars, and pink triangles for hemostasis and wound healing; and subsequent healed and remodeled tissue.

Traditional wound dressings, such as gauzes and bandages, are inexpensive and easy to use; however, they are ineffective for covering deep or irregular wounds or for providing a moist environment, which can cause pain and secondary tissue damage upon removal [38,39]. Hydrogel dressings, which have high water-retention capacity and extracellular matrix (ECM)-mimicking properties that allow gas exchange, have been widely used as advanced dressings [40,41,42]. Their hydrophilicity and adhesiveness enable close contact with wounds, seal bleeding sites, and protect against secondary infections, while providing a moist wound environment that promotes tissue regeneration and reduces pain during removal [40,43]. Nevertheless, conventional hydrogel dressings are mechanically weak and typically patch- or sheet-type materials, limiting their use for deep or irregularly shaped wounds. In contrast, IHs can rapidly fill deep, irregular, and complex-shaped wounds, promote faster hemostasis, and enable localized delivery of bioactive agents (Figure 3). They can also degrade naturally or remain in situ until absorbed, avoiding discomfort and secondary injury. Rheological parameters during injection can influence the internal network of the administered IHs, thereby determining therapeutic efficacy. Therefore, IHs for hemostasis and wound healing represent promising next-generation biomaterials compared with traditional dressings and conventional hydrogel dressings.

Li et al. [39] developed N,O-carboxymethyl chitosan/oxidized chondroitin sulfate (N,O-CMC/OCS) IHs under physiological conditions by varying the mass ratio of CMC:OCS (m__CMC:OCS_ = 2:1, 1:1, and 1:2) through a Schiff base reaction between the amino groups of N,O-CMC and aldehyde groups of OCS. The N,O-CMC/OCS) IHs exhibited a high equilibrium swelling ratio (*q*_e_, 10~20), good degradability (50~60% within 2 weeks), nontoxicity, antibacterial activity due to electrostatic membrane disruption, and outstanding hemostatic and wound healing efficacy in a mouse liver model. The *G*′ values of N,O-CMC/OCS IHs remained higher than the G″ values after gelation, which was likely attributed to the covalent bonds formed between N,O-CMC and OCS through Schiff-base reactions. From the time-sweep measurements, the gelation time (*t_gel_*) of CMC/OCS IHs with m__CMC:OCS_ = 2:1 was determined to be about 133 s, whereas the other IHs with m__CMC:OCS_ = 1:1 or 1:2 showed about 5 s. Thus, gelation rate and modulus were key rheological parameters influencing the dressing applicability. Similarly, Deng et al. [42] developed self-healing IHs based on adenine-modified chitosan (AC) derivatives by varying the molar ratio of 3-(9-adeninyl)-propionic acid (A-COOH) to N-acetylglucosamine unit (AGU) (M_ACOOH:AGU_) and by adjusting the concentration of AC through a simple heating/cooling process. The AC IHs exhibited excellent hemostatic and wound-healing efficacy, as well as antibacterial activity. The frequency-sweep measurements (*f*: 1–100 rad/s) showed a stable crosslinked structure, with *G*′ > *G*″, over the entire frequency range. In addition, *G*′ values decreased with increasing the M_ACOOH:AGU_, indicating a decrease in crosslinking density due to weaker hydrogen-bonding interactions between the amino and hydroxyl groups of chitosan as the degree of adenine substitution (DS) increased. In contrast, they increased with higher AC concentrations, suggesting an enhancement in crosslinking density. Moreover, the AC IHs exhibited shear-thinning behavior, with lower *η* at higher M_ACOOH:AGU_ and lower AC concentrations, indicating improved injectability. In temperature-sweep measurements, the *G*′ values of IHs decreased with increasing *T* from 10 °C to 80 °C and recovered upon cooling, indicating excellent reversible thermal stability. In strain sweep measurements (*γ*: 0.1–1800%), a crossover point of *G*′ and *G*″, indicating the sol-gel transition, appeared at *γ* = 680%. The self-healing behavior of the IHs was confirmed by alternate step-strain tests (*γ*: alternating between 10% and 1200% with 200 s intervals). In a different crosslinking approach, Yang et al. [44] fabricated multifunctional IHs composed of thiol-modified poly(γ-glutamic acid) (γ-PGA-SH) and oxidized hyaluronic acid (HA-CHO) at different concentrations (5, 10, and 15 wt%) via a thiol–aldehyde addition reaction. With increasing concentration, the *t_gel_* of the IHs shortened, while the modulus and compression stress increased, owing to additional hydrogen bonding and physical entanglement of γ-PGA and HA chains. The IHs showed high water content (85–95%), significant antioxidant activity, excellent tissue adhesiveness, complete in vivo degradation within 3 days, and significantly enhanced wound-healing efficacy in a full-thickness skin defect model. The time-sweep measurements showed that the sol-gel transition occurred within approximately 3 min. In the amplitude-sweep measurements (*γ*: 0.1–1000%), all IHs exhibited *G*′/*G*″ > 10 over a wide linear viscoelastic (LVE) region, indicating stable elastic-network formation. The frequency-sweep measurements (*f*: 0.1–100 Hz) showed that a lower polymer concentration produced smaller G′ and more pronounced frequency dependence, indicating a less stable network structure. Moreover, the IHs displayed shear-thinning behavior and a distinct stress-relaxation feature, with lower concentrations corresponding to faster polymer relaxation, enabling excellent injectability and adaptability to irregular wound sites. Another biocompatible IH system presented by Xie et al. [45] consisted of carboxymethyl cellulose (CMC) and europium-ethylenediaminetetraacetic acid (Eu-EDTA) complex. With increasing concentration, the *q*_e_ of the CMC-Eu-EDTA IHs decreased, indicating a higher crosslinking density. The hydrogel network was stabilized by dynamic, reversible Eu^3+^–carboxylate coordination bonds, resulting in a self-healing 3D structure. The intrinsic fluorescence of Eu^3+^ enabled real-time pH monitoring over 4.5–7.5, enabling visible detection of wound status. In addition, Eu^3+^-induced reactive oxygen species (ROS) generation promoted angiogenesis. The dynamic bonds endowed the IHs with injectability and self-healing, enabling complete structural recovery within 10 min after mechanical damage. Moreover, the IHs exhibited both therapeutic and diagnostic capabilities for wound care. The *G*′ values of all IHs remained higher than G″ across *f* = 0.1–100 rad/s, indicating a stable viscoelastic network with solid-like behavior. With increasing Eu-EDTA concentration, the *G*′ value increased from 81.3 ± 2.2 Pa to 236.7 ± 10.0 Pa, presenting enhanced coordination-interaction-driven crosslinking density. Furthermore, shear-thinning behavior was observed over $\overset{\cdot }{\gamma }$ = 0.1–100 s^−1^, demonstrating great injectability. The sol-gel transition points occurred at *γ* = 300–1200%, and both *G*′ and *G*″ values recovered quickly to their original values during cyclic step-strain tests (alternating between 1% and 1300%), demonstrating excellent reversible network reconstruction. Likewise, Shi et al. [46] developed an effective IH hemostatic sealant by reacting poly(ethylene imine) (PEI) and adipic dihydrazide (ADH) with a 4-arm polyethylene glycol (PEG) thioester-aldehyde crosslinker through Schiff-base condensation. The thioester linkages within the IHs enable on-demand dissolution via a thiol–thioester exchange reaction upon exposure to an L-cysteine methyl ester (CME) solution. Additionally, the covalent interactions of aldehyde end groups with tissue amino groups enhance tissue adhesion in IHs. The dynamic imine bonds and reversible thioester exchange provided tunable mechanical robustness, viscoelasticity, and biodegradability in the IHs. The *t_gel_* of the IHs shortened with increasing PEI or 4-arm PEG crosslinker concentration and decreasing ADH concentration. The *G*′ values of IHs remained higher than *G*″ across the entire frequency range, indicating stable viscoelastic solid-like behavior, while shear-thinning and high compressive resilience supported injectability and adaptability to irregular wound shapes. The addition of ADH decreases adhesion force by consuming aldehyde groups. It also reduces brittleness, owing to its inherent nature, and improves flexibility and mechanical stability. In another related example, Fang et al. [47] prepared a bioinspired multifunctional IH (CQCS@gel) composed of catechol-modified quaternized chitosan (CQCS) and dibenzaldehyde-terminated PEG (DB-PEG-CHO) via Schiff-base click chemistry for hemostasis and infectious wound care. Additional catechol groups formed hydrogen bonding and π-π interaction, which enhanced tissue adhesion. In addition, the reversible imine linkage provided pH-responsive drug release and self-healing behavior. This hybrid network produced stable and suitable IHs for wound care. Additionally, CQCS@gel demonstrated excellent biocompatibility, antioxidant ability, broad-spectrum antibacterial activity, rapid hemostasis, and effective wound closure. The *t_gel_* of the IHs was less than 30 s at CQCS concentrations above 1.5 wt%. The time-sweep tests revealed that both *G*′ and *G*″ remained nearly constant over 600 s, with *G*′ consistently higher than *G*″, indicating the elastic-dominant behavior and structural stability of the IHs. With increasing CQCS concentration, the *G*′ values of the IHs gradually increased, indicating enhanced crosslinking density arising from ionic and hydrogen-bonding interactions. The *G*′ of damaged IHs as a function of *f* progressively recovered to their initial levels over the healing period, confirming reversible network reconstruction and strong self-healing capability.

For optimal hemostatic performance, IHs require extremely fast gelation (<10 s), low injection resistance, sufficient *G*′ to resist blood flow without impairing tissue compliance, and rapid structural recovery. Despite significant advancements, many reported hydrogels still lack in vivo validation, and discrepancies between in vitro and in vivo modulus and deformation remain unsolved. Continued development must incorporate additional physiology-related evaluations to ensure reliable sealing strength and durability under dynamic bleeding conditions.

### 2.2. Tissue Engineering and Regenerative Medicine Scaffolds

**Figure 4:**
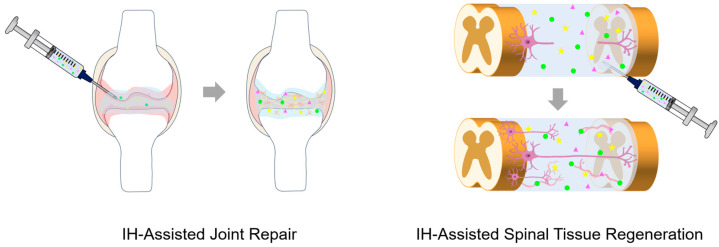
Schematic illustration of the IH administration, with bioactive ingredients represented by the green dots, yellow five-pointed stars, and pink triangles for scaffold formation promoting joint repair and spinal tissue regeneration as representative applications in tissue engineering and regenerative medicine.

IH scaffolds have emerged as versatile biomaterials that enable minimally invasive implantation and in situ formation of a 3D network, adapting to the irregular defect geometries (Figure 4). These scaffolds mimic the structural and biochemical features of the extracellular matrix (ECM) and provide a hydrated and bioactive environment that supports cell adhesion, proliferation, and differentiation, while facilitating host cell integration and promoting tissue regeneration [48]. Recent IH scaffolds commonly employ a variety of synthetic and natural polymers, such as poly(lactic acid) (PLA), Poly(lactic-co-glycolic acid) (PLGA), poly(trimethylene carbonate) (PTMC), PEG, alginate, chitosan, collagen, and HA due to their excellent biocompatibility, injectability, tunable mechanical strength, degradability, and ability to support cells. Moreover, bioactive and therapeutic agents are frequently incorporated into IH scaffolds to endow them with additional biological functions [49,50,51]. Regenerative medicine is an interdisciplinary field aiming to repair or replace damaged tissues and organs. It integrates tissue engineering, stem cell science, gene therapy, and bioactive material delivery to promote endogenous healing and restore physiological function, along with emerging fields such as exosome therapy, immunomodulatory regeneration, and organoid technology [52]. Unlike traditional therapies that only alleviate symptoms or replace damaged tissues, regenerative medicine distinctively stimulates natural healing ability and intrinsic repair mechanisms to enable functional recovery [53]. In this context, IHs represent an ideal type of biomaterials because their fluidity allows in situ gelation and minimally invasive application to irregular defect sites, while providing a supportive microenvironment for cell engraftment, growth factor delivery, and tissue regeneration [54]. Furthermore, their viscoelasticity, porosity, and degradation kinetics can be precisely tuned to meet the distinct requirements of specific tissues [55]. Accordingly, IH-based scaffolds have been successfully applied in tissue engineering and regenerative medicine, including bone and cartilage repair, and the regeneration of retinal, oral, neural, and muscle tissues [48,49,50,51,56,57,58].

Shi et al. [48] developed an injectable chitosan/polyethylene glycol–silicotungstic acid (CS/PEG-SiW) double-network hydrogel fabricated via dual dynamic interactions for cartilage tissue engineering. These IHs were fragmented into micro-sized particles (47.6–63.8 μm), injected, and then recombined into a stable form via electrostatic interactions and a Schiff-base reaction among CS, SiW, and PEG-DA. These CS/PEG-SiW IHs exhibited excellent mechanical strength, good biodegradability (approximately 51% within 21 days), cytocompatibility, and effective promotion of cartilage regeneration. The flow tests showed that the CS/PEG-SiW IHs exhibited shear-thinning behavior, indicating excellent injectability. Frequency-sweep measurements showed that the CS/PEG-SiW IHs exhibited predominantly elastic behavior and higher *G*′ than the CS/PEG IHs, owing to dual dynamic crosslinking via Schiff base and electrostatic interactions. An increase in SiW concentration from 1.0 wt% to 2.0 wt% significantly increased *G*′ and compressive strength, confirming higher crosslinking density. Recombined porous hydrogels displayed lower *G*′ and compressive modulus than bulk hydrogels; however, they exhibited higher maximum strain, demonstrating excellent flexibility and self-healing. The CS/PEG-SiW IHs provided an optimal balance between mechanical stability and injectability, making them suitable for cartilage tissue-engineering applications. Similarly, Huang et al. [49] reported the development of three rapid in situ-forming IHs for the treatment of intervertebral disc (IVD) degeneration. Methacrylate chitosan (CSMA) IHs were fabricated via photo-crosslinking, the CSMA and aldehyde polyethylene glycol (PEGDA) (CSMA-PEGDA) IH via the Schiff base reaction between CSMA and PEGDA, and the CSMA-PEFDA-L IHs through both Schiff base and photo-crosslinking mechanisms. CSMA-PEGDA-L IHs demonstrated high compressive strength, low cytotoxicity, excellent biodegradability, and excellent therapeutic efficacy. The differences in *G*′ among CSMA1-PEGDA, CSMA2-PEGDA, and CSMA3-PEGDA mainly originated from the variation in CSMA concentration of 1, 2, and 3 wt%. An optimal 2 wt% CSMA provided the highest crosslinking efficiency and the most stable hydrogel network. Lower CSMA concentration led to a weaker gel due to reduced Schiff-base reaction, and higher CSMA concentration resulted in lower mixing and crosslinking efficiency due to higher *η*. CSMA-PEGDA-L dual-network IHs with appropriate CSMA content represent an excellent therapeutic material system for intervertebral disc repair. In a different material strategy, Liu et al. [50] proposed a tissue-adhesive IH for treating inner retinal tissue injuries. A cinnamic acid (CA)-grafted gelatin (Gel) (GelCA) IH was prepared by photo-crosslinking, and its nanocomposite forms, polydopamine (PDA)-loaded GelCA IHs (PDA@GelCA) and curcumin (Cur)-loaded PDA nanoparticles (NPs) incorporated into GelCA IHs (Cur@PDA@GelCA) were fabricated through π–π stacking and hydrogen bonding. This composite hydrogel exhibited excellent biocompatibility and antioxidant properties, as well as enhanced tissue adhesion. The *G*′ of the GelCA IHs remained higher than *G*″, indicating stable elastic behavior before and after injection. With increasing GelCA concentration, the *t_gel_* required for structural stabilization increased, and both *G*′ and the compressive modulus increased, reflecting enhanced stiffness due to higher crosslinking density. The incorporation of PDA and Cur@PDA NPs further increased *G*′ through NP-matrix interactions. Cur@PDA@GelCA IHs exhibited shear-thinning behavior, reflecting excellent injectability, and degraded by approximately 80% in collagenase within 8 h. The Cur@PDA@GelCA IHs have significant potential to promote neuroregeneration. Extending these concepts toward dental and periodontal applications, Atila et al. [51] prepared low-molecular-weight chitosan (LCH) and high-molecular-weight chitosan (HCH) IHs containing curcumin (Cur) and α-tocopherol (Toc)-loaded liposomes (Lip/Cur + Toc). Lip/Cur + Toc@LCH and Lip/Cur + Toc@HCH IHs exhibited excellent water absorption capacity, sustained drug release properties (10–25% for three weeks), antibacterial activity, and excellent wound-healing efficacy using human gingival fibroblasts (hGFs) and human dental pulp stem cells (hDPSCs). In the flow tests, LCH and Lip/Cur + Toc@LCH IHs showed lower *η* than HCH and Lip/Cur + Toc@HCH IHs, respectively, due to their lower molecular weight. Both IHs exhibited shear-thinning behavior, confirming their excellent injectability. HCH and Lip/Cur + Toc@HCH IHs showed higher *G*′ and *G*″ and a wider LVE region than LCH and Lip/Cur + Toc@LCH IHs, respectively. As the temperature increased from 24 °C to 40 °C, *η* increased. Additionally, the addition of Lip/Cur + Toc decreased *G*′, *G*″, and *η*, suggesting that Lip/Cur + Toc-loaded LCH and HCH IHs have higher injectability. Yi et al. [56] engineered an electroconductive IH for peripheral nerve regeneration and motor function restoration by crosslinking aldehyde-based hyaluronic acid (ALHA) and carboxymethyl chitosan (CMCS)-grafted polyaniline (PANI) (CP), termed the ALHA/CMCS/CP (ACCP) hydrogel. The ACCP hydrogels were formed via Schiff-base reactions between ALHA aldehyde groups and CP amino groups, and via electrostatic interactions between ALHA and PANI, resulting in a dynamically crosslinked network with reversible interactions. The ACCP hydrogels exhibited excellent biocompatibility, self-healing behavior, and degradability, tunable electrical conductivity (up to 8.27 × 10^−4^ S cm^−1^), and proper elastic modulus of 33.5–66.8 kPa, comparable to that of native sciatic nerve tissue. The ACCP(CP3) hydrogel significantly enhanced axonal remyelination, nerve conduction, and muscle recovery, indicating its high efficacy for peripheral nerve regeneration. The ACCP hydrogel displayed predominant elastic behavior with *G*′ consistently higher than *G*″ over the range of 1–100 rad/s. *G*′ values increased with higher CP concentrations, suggesting that the incorporation of CP enhanced the crosslinking density and thereby improved the stiffness of the conductive hydrogels. The critical strain at network failure was measured to be 377.2% in amplitude-sweep tests (0.1–1000%), indicating high structural resilience. Step-strain tests confirmed full recovery of *G*′ at 1% after high-strain (500%) disruption, demonstrating excellent self-healing performance due to the dynamic crosslinks. Shear-thinning behavior further verified its injectability and dynamic bond reversibility. Likewise, Li et al. [57] introduced a rapid-gelling, self-healing hyaluronate IH for treating spinal cord injury. The IH was prepared via an in situ Schiff-based reaction between aldehyde-modified hyaluronic acid (AHA) and 3,3′-dithiobis(propionyl hydrazide) (DTP), producing both arylhydrazone and disulfide dynamic covalent bonds, resulting in a dual crosslinked AHA/DTP IH system. The IH exhibited a rapid sol-gel transition within 30 s and complete gelation within 50 s, displayed stiffness values (2–20 kPa) comparable to neural tissue, and maintained structural stability under physiological conditions, while degrading rapidly in acidic environments. Moreover, the IH demonstrated no cytotoxicity toward neural stem cells (NSCs), and the scaffolds enhanced neuronal differentiation with elevated Yuj1 and NF-200 expression and promoted angiogenesis, remyelination, and neuronal regeneration. Frequency sweep tests showed that *G*′ was higher than *G*″ across 0.1–10 rad/s, indicating solid-like behavior and excellent structural stability. Step-strain recovery tests from 1% to 450% showed complete self-healing within 1 h, enabling the IH to withstand external tensile forces. This property is attributed to the dynamic bonds mentioned above, with additional contributions from hydrogen bonding. Furthermore, the AHA/DTP hydrogel exhibited shear-thinning behavior, confirming its excellent injectability. Gilan et al. [58] developed self-healing HA-based IHs physically crosslinked with pytic acid (PA), in which the weight ratio of HA to PA (3.3:1–6.6:1) was varied to optimize the network properties of the resulting HAPA IHs for regenerative medicine applications. The HAPA IHs behaved solid-like at the HA:PA weight ratio in the range of 3.3:1–6.6:1. The HAPA45 IHs with the optimal HA:PA weight ratio (4.5:1) produced strong and homogeneous IHs with superior mechanical strength, injectability, and self-healing efficiency. The IHs exhibited a porous, interconnected microchannel structure (pore size ~25 μm), which is favorable for nutrient diffusion and cell proliferation. Moreover, the IHs showed excellent biocompatibility, stable tissue adherence, and enhanced cell proliferation by ~14% after 72 h. The HAPA IHs exhibited shear-thinning and thixotropic behavior, with decreasing *η* as shear rate increased from 10^−3^ to 10^3^ s^−1^, confirming excellent injectability. The HAPA45 IHs showed the highest zero-shear viscosity (*η*_0_ = 64.85 Pa·s), the longest relaxation time (*t*_R_ = 31.65 s), and a solid-like viscoelasticity (*G*′ > *G*″), which is likely due to the greater number of hydrogen bonds forming a stronger physical network. In cyclic strain tests (1–3000%), HAPA45 IHs rapidly recovered their structure after high strain (3000%), possibly due to the cooperative self-assembly of HA and PA via dynamic hydrogen bonds, providing a reversible sol-gel transition and structural adaptability suitable for injectable regenerative medicine scaffolds.

Ideal scaffold rheology generally requires moderate to high *G*′ for defect support, a broad LVE region to withstand physiological deformation, and controlled stress-relaxation behavior to facilitate cell infiltration and tissue remodeling. Although IH scaffolds exhibit tunable viscoelastic properties, inconsistent or insufficient in vivo rheological data, and limited analysis of long-term in vivo stiffness evolution hinder direct comparison across studies. Continued progress requires standardized rheological protocols and more evident mechanistic correlations between rheological properties and regenerative performance.

### 2.3. Drug Delivery Systems (DDSs)

**Figure 5:**
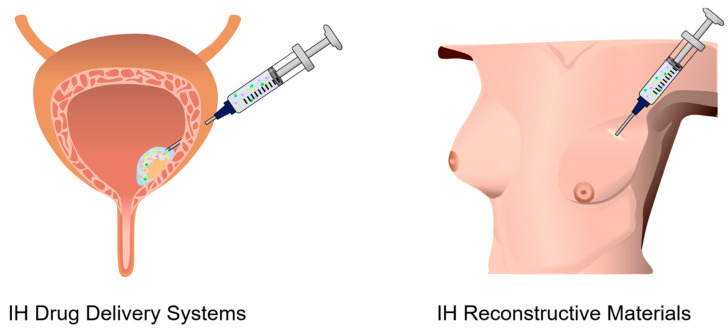
Schematic illustration of the local injection of an IH with bioactive ingredients represented by the green dots, yellow five-pointed stars, and pink triangles for bladder-lesion treatment and breast-tissue reconstructive augmentation as representative DDSs and reconstructive materials, respectively.

The use of drug delivery systems (DDSs) has been a central strategy in modern biomedicine for treating a broad spectrum of diseases by enabling precise spatial and temporal control over therapeutic release, thereby enhancing efficacy while minimizing systemic toxicity. Effective DDS requires carrier materials that possess biocompatibility, exhibit excellent injectability, and have high drug loading and release efficiency [59]. Hydrogels can be ideal carriers not only because of their tunable porosity and 3D crosslinked network structure, which can encapsulate, transport, and release diverse therapeutic agents, such as proteins, nucleic acids, and cells, but also because of their tunable swelling, viscoelastic, and mechanical properties [60,61]. In recent years, IHs with fluidity have attracted attention among various DDS platforms because they offer in situ gelation, adaptability to irregular sites, and the ability to provide localized and sustained drug release, enhancing therapeutic efficacy (Figure 5, left) [62,63,64]. Modulating their rheological characteristics enables fine control of gelation kinetics, structural integrity, viscoelasticity, and mechanical stability, thereby optimizing injectability and therapeutic performance [65]. In particular, IHs facilitate minimally invasive drug delivery and promote targeted drug accumulation within tumor microenvironments via the enhanced permeability and retention (EPR) effect, achieving enhanced therapeutic efficacy while minimizing systemic exposure [59,66].

Liu et al. [62] reported an interpenetrating polymer network (IPN) IH that heals and remodels the microenvironment in bone erosion associated with rheumatoid arthritis (RA). The click reaction between hyaluronic acid-tetrazine (HA-Tz) and collagen-norbornene (Col-Nb) produced the CH hydrogels. Additionally, bisphosphonate-functionalized hyaluronic acid macromers (HABPs) and zinc-doped biphasic calcium phosphate (ZnBCP) nanorods were incorporated via electrostatic interactions, leading to the formation of the HABP-ZnBCP@CH IPN IHs. The IPN IHs exhibited excellent adaptability to defect shape, multi-ion delivery, strong anti-inflammatory properties, strong bone-regenerative effects, and excellent biodegradability. The frequency-sweep results showed predominantly elastic behavior across the entire range, indicating their predominant elastic property. Additionally, the HABP-ZnBCP@CH IHs showed higher *G*′ than the CH hydrogels, owing to the reinforced IPN structure and synergistic interactions among HABP, ZnBCP, and CH. In a related strategy for neural repair, Qi et al. [63] presented a dual-drug IHs (NSCs-cfGel) formed by aldehyde-modified oxidized dextran (o-Dex) and hydrazide-functionalized four-arm polyethylene glycol (4-arm-PEG-NHNH_2_) via reversible hydrazone crosslinking, encapsulating neural stem cells (NSCs). The NSCs-cfGel was loaded with two drugs, cetuximab and FTY720, for the treatment of spinal cord injury (SCI). Cetuximab promotes neuronal differentiation, and FTY720 inhibits glial scarring, thereby enhancing neural regeneration. The NSCs-cfGel hydrogel exhibited high biocompatibility, tunable elasticity, and a porous structure favorable for cell proliferation. In vivo transplantation of IHs into a rat model of complete spinal cord transection resulted in enhanced axonal regeneration and locomotor recovery. The IHs formed rapidly within approximately 2 min at 37 °C and exhibited stable viscoelastic behavior with *G*′ consistently higher than *G*″, confirming a predominantly elastic nature. The *G*′ remained below 7 kPa, which was suitable for the proliferation and differentiation of NSCs. Besides, the time-dependent *η* and the fracture stress were determined to be 600 Pa·s and approximately 4200 Pa, respectively, which can provide stable adherence to the rat spinal cord. In vitro, the NSCs-cfGel maintained structural stability for up to 5 days before gradually degrading, whereas the IHs remained stable for the first 2 weeks and degraded by 18 days in vivo. The IHs exhibited a sustained drug release, with approximately 83% release within 36 h, followed by saturation. This controlled release behavior suggests the potential of the NSCs-cfGel as a drug-delivery system to improve neural regeneration. In another injectable DDS design, Li et al. [64] developed a multifunctional composite IH delivery system to achieve high drug loading efficiency and prolonged local anesthesia release. The CMHA IH matrix (Gel) was formed via Schiff-base crosslinking between oxidized hyaluronic acid (OHA) and modified carboxymethylcellulose (CMC-ADH). Ropivacaine (ROP)-loaded methacryloyl HA (HAMA)-based hydrogel core–shell microspheres (HMS-ROP, 200–400 μm in size) and the sedative dexmedetomidine (DEX) were loaded into the Gel IHs, producing Gel/HMS-ROP/DEX composite DDS. The composite IHs exhibited self-healing, strong tissue adhesion, and mechanical resilience. Additionally, their *t_gel_* increased to ~35 s, and porosity decreased with increasing HMS-ROP content. Moreover, the composite IHs showed superior injectability, biocompatibility, controlled degradation, a high ROP loading capacity (≈77%), and sustained drug release for up to 40 h. The composite IHs displayed *G*′ consistently greater than *G*″ over the entire strain range, indicating an elastic-dominant network structure and shear-thinning behavior, confirming excellent injectability. Under cyclic strain from 1–200%, the structure collapsed at 200% and recovered after a low strain of 1% was applied, demonstrating self-healing after injection. Gel/HMS-ROP demonstrated sustained drug release for more than 168 h, resulting in extended local anesthetic effects. Overall, Gel/HMS-ROP composite IHs represent a promising DDS for sustained drug release. Likewise, Park et al. [65] reported an injectable thermosensitive deferoxamine (DFO) NP-loaded crosslinked HA (xHA) incorporated into Pluronic F127 hydrogels, designed for sustained DFO release to treat patients with iron overload. The DFO NPs (~5.2 nm) were uniformly dispersed within the xHA/F127 IHs, providing a stable, biocompatible platform for prolonged chelator delivery. The DFO-NP/HA/F127 IHs exhibited rapid gelation at 37 °C, demonstrating excellent injectability, minimal initial burst release, and robust structural integrity. In vivo fluorescence imaging showed sustained and prolonged release of DFO for more than 14 days, resulting in a 4.3-fold increase in half-life compared with free DFO. The IHs displayed a distinct thermosensitive sol-gel transition around 30 °C by a micellar mechanism, accompanied by a pronounced increase in the *G*′ beyond the critical gelation temperature (*T_gel_*). Their *G*′ was higher than *G*″ over the temperature range of 5–40 °C, and *G*′ increased up to ~8500 Pa. They also showed shear-thinning behavior, facilitating injection without structural disruption. These rheological features ensured reversible sol-gel transition, stable mechanical integrity, and sustained drug release under physiological conditions. Extending DDS-IH to cancer immunotherapy, Kuwentrai et al. [66] studied an injectable hydrogel-based drug formulation that enhances the efficacy of cancer immunotherapy and stimulates the formation of tertiary lymphoid structures (TLSs) that recruit B and T cells and promote mature TLS formation in the tumor microenvironment through localized co-delivery of the immunomodulatory proteins CXCL13 and LIGHT proteins. A drug delivery IH system (HA-CPP⊂CB) was prepared by conjugating HA with cationic cell-penetrating peptides (CPP, 4-(4-chlorophenyl)pyridine) via host-guest interactions between HA-CPP and a macrocyclic host molecule, cucurbit [8] uril (CB [8]), forming a dynamic supramolecular network with reversible interactions. These HA-CPP⊂CB IHs exhibited biocompatibility, shear-thinning behavior, confirming excellent injectability, tunable porosity, reversible self-healing, and localized and sustained cytokine release. In a B16-OVA melanoma mouse model, sustained delivery of CXCL13 and LIGHT induced mature TLS formation, enhanced antigen-specific T-cell activation, and significantly inhibited tumor growth. Combining therapy with anti-PD1 antibodies further improved survival and immune infiltration, demonstrating synergistic enhancement of tumor immunotherapy. The HA-CPP⊂CB IHs exhibited frequency-dependent viscoelasticity with *G*′ exceeding *G*″, demonstrating elastic dominance and stable network formation, with the *t*_R_ of approximately 1 min. The IHs also showed dynamic and reversible behavior, enabling self-healing. Cyclic step-strain tests (alternating between 1% and 100% at 10 rad/s) confirmed that *G*′ quickly recovered after high strain (100%) was applied, confirming the excellent self-healing properties due to dynamic host–guest interactions. These rheological properties provided well-controlled injectability, mechanical stability, and sustained release under physiological conditions, which are desirable features for a DDS. Finally, Wu et al. [67] designed a thermosensitive IH composed of triblock copolymer PLGA–PEG–PLGA for co-delivery of cetuximab (Cmab) and the endocytosis inhibitor prochlorperazine (PCZ), termed Gel@Cmab/PCZ, to enhance antibody-dependent cellular cytotoxicity (ADCC) in colorectal cancer (CRC). The Gel@Cmab/PCZ IHs exhibited biocompatibility, biodegradability, and sustained and localized drug release of 70–80% over more than 15 days. In both subcutaneous and orthotopic CRC models, combination treatment markedly suppressed tumor growth, prolonged survival, and enhanced NK-cell infiltration compared to free-drug administration. The Gel@Cmab/PCZ IHs showed a sol-gel transition near 30 °C and shear-thinning behavior, enabling smooth injection and homogeneous drug dispersion. These rheological properties supported easy administration, provided structural stability, and enabled sustained drug release at the tumor site.

Effective DDS-IHs ideally maintain low *η* for injectability, moderate *G*′ to preserve depot structure, and predictable time-dependent rheology that correlates with controlled diffusion. While rheological modulation enables controlled drug release, many studies provide insufficient quantitative relationships between viscoelastic behavior and release kinetics. Additionally, in vivo swelling and *G*′ changes are challenging to quantify and often overlooked. More comprehensive rheology–release correlation models are needed to guide predictable therapeutic performance.

### 2.4. Reconstructive Materials

Reconstructive surgery aims to restore both structure and function of damaged or lost tissues due to trauma, tumor resection, congenital anomalies, or infection [68]. Traditional reconstructive approaches, including autologous grafts and solid implants, are constrained by donor-site morbidity, limited adaptabiity to irregular defect geometries, and significant surgical invasiveness with associated complications [69,70]. IHs have emerged as promising alternative material systems owing to their minimal invasiveness, tunable mechanical properties, ability to conform to complex geometries, and biocompatibility with host tissues [71]. Recent formulations utilize biocompatible polymers, such as collagen, cellulose, hyaluronic acid (HA), PEG, poly(N-isopropylacrylamide) (PNIPAM), and poly(2-hydroxyethyl methacrylate) (PHEMA), and have been applied for cleft palate and craniofacial bone repair, breast augmentation, and soft-tissue regeneration following tumor excision (Figure 5, right) [72,73,74,75,76]. IHs exhibit viscoelastic properties comparable to those of native soft tissues, as well as shear-thinning and rapid self-recovery behaviors, facilitating smooth injection through fine needles while maintaining shape retention after implantation [73,74]. These rheological features are essential for functional tissue reconstruction.

Kambhampatiet et al. [72] developed a photo-crosslinkable IH system, formed by mixing a methacrylate-functionalized hydroxyl polyamidoamine dendrimer (D-MA) and methacrylated HA (HAMA) (OcuPair^TM^), for sealing and repairing traumatic corneal rupture. The formulations (mixing ratios of D-MA and HAMA: 70:30, 50:50, and 30:70) exhibited biocompatibility, high transparency, tunable *η* (0.1 × 10^3^–8.0 × 10^3^ cP), and in situ gelation within 90 s under UV irradiation. The optimal formulation (30:70) produced a flexible, viscous, and biocompatible material that adhered to wet ocular surfaces and withstood intraocular pressure (>80 mmHg) without leakage. In vivo studies in rabbit and porcine models demonstrated effective closure of linear, stellate, and circular corneal wounds with minimal inflammation. The formulation (30:70) exhibited η of 5.0 × 10^3^–7.0 × 10^3^ cP, which decreased to 1.0 × 10^3^ cP with increasing $\overset{\cdot }{\gamma }$ from 0.1 s^−1^ to 100 s^−1^. This shear-thinning behavior allowed extrusion through a 30G gauge needle. In addition, the formulation (30:70) showed rapid gelation (10–30 s) and G′ > G″ across the frequency range, indicating elastic dominance. These rheological features ensured easy handling for ocular applications. When applied to a porcine cornea, the IHs formed by UV irradiation sealed and fixed the incised corneal tissue without causing edema or degradation. Furthermore, the hydrogel maintained a hydration level of 78–88%, comparable to that of the human cornea. Extending reconstructive hydrogel strategies to soft-tissue implants, Vashahi et al. [73] suggested injectable IHs with viscoelastic and mechanical properties similar to those of soft tissues, such as adipose and brain tissue, for reconstructive implants. A linear-bottlebrush-linear (LBL) copolymeric IH was prepared with PEG as the thermosensitive bottlebrush block part and PNIPAM as the linear block part. The LBL structure was self-assembled at 37 °C via physical crosslinking and microphase separation of the PNIPAM domain. The *T*_gel_ depended on the LBL composition and concentration and increased with increasing concentration and decreasing PNIPAM linear block size, due to reduced hydrophobic interactions. The LBL IHs showed high elasticity, reversible self-healing behavior, a unique J-shaped stress–strain curve analogous to that of soft tissues such as fat and brain, and prevented water expulsion during gelation. The *G*′ value depended on the LBL concentration and composition, increasing from 10 Pa to 10^5^ Pa as concentration and bottlebrush size increased. The IHs withstood strains up to 700% and rapidly reassembled after rupture. The IHs exhibited excellent biocompatibility (80–90% fibroblast viability), minimal inflammation, and adaptability for reconstructive applications. Therefore, this LBL IH is proposed as a body filler for reconstructive surgery. In another multifunctional reconstructive approach, Yang et al. [74] developed a photothermal hybrid IH system composed of methylcellulose, indocyanine green (IR820), and porous PLGA microspheres (MPs), termed IR820/Mgel, to prevent tumor recurrence and reconstruct breast defects following breast cancer surgery. The IR820 served as a near-infrared (NIR) light-absorbing material to generate localized hyperthermia (>50 °C) under NIR irradiation, eliminating 4T1 tumor cells and preventing post-surgical tumor recurrence in vivo. The MPs improved mechanical strength and structural integrity and enhanced cell adhesion for breast reconstruction. The hybrid IR820/Mgel IHs exhibited excellent biocompatibility, sustained photothermal activity, and long-term shape retention after injection. The hybrid IHs displayed a sol-gel transition temperature of 29–31 °C and rapid gelation (95 s for pure gel and 118 s for Mgel containing MPs), and their *G*′ surpassed *G*″ with increasing temperature, indicating a progressively strengthened network structure. Therefore, this photothermal hybrid IH represents a promising material system for breast reconstruction. Similarly, Wang et al. [75] fabricated a transgenic silkworm system to biosynthesize an engineered growth factor-β1 (eTGF-β1)-functionalized silk sericin IH (eTGF-β1 SH) for repairing alveolar bone defects (ABDs) associated with cleft palate. eTGF-β1 improved ECM-binding efficiency, enhancing the biological activity of the sericin matrix. The eTGF-β1 SHs were porous and biocompatible, formed via mild aqueous processing and physical gelation. They exhibited sustained release of eTGF-β1 for over 30 days, promoting fibroblast proliferation, osteoblast maturation, and expression of osteogenic markers. In vivo studies using a rabbit alveolar cleft model demonstrated that the eTGF-β1 SHs provided complete bone regeneration with mature collagen I deposition and dense trabecular formation. The eTGF-β1 SHs showed shear-thinning and elastic-dominant behavior, ensuring injectability and network stability. β-sheet crystallinity (~41%) contributed to mechanical robustness and shape integrity. These rheological properties support the viscoelasticity and mechanical integrity required for bone-tissue reconstruction. He et al. [76] introduced a next-generation breast implant material based on Fe^3+^ Coordinated poly(2-hydroxyethyl methacrylate (PHEMA)/maleic acid(MA) (PHM/Fe^3+^) IHs to overcome the limitations of conventional silicone implants, such as hydrophobicity and capsular contracture. PHM/Fe^3+^ IHs showed excellent toughness (~1 MJ m^−3^) and fatigue resistance after 1000 compression cycles, because mechanical stress was dissipated while maintaining elasticity similar to soft tissues. In vivo tests demonstrated minimal inflammation and significantly thinner fibrous capsules than smooth and textured silicone implants. Besides, the PHM/Fe^3+^ IHs exhibited that *G*′ was consistently greater than *G*″ across an angular frequency range of 1–100 rad s^−1^, and *G*′ increased with increasing angular frequency, indicating a solid-like behavior. Incorporation of Fe^3+^ ions increased the apparent activation energy (E_a_ ≈ 57 kJ mol^−1^), reflecting enhanced network stability and restricted molecular mobility. The PHM/Fe^3+^ IHs displayed shear-thinning behavior and rapid self-recovery after deformation, supporting excellent injectability and mechanical adaptability.

Ideally, reconstructive IHs require intermediate *G*′ to balance shape retention with flexibility, sufficiently high *τ*_y_ to prevent migration, and rapid post-injection recovery to conform stably to defect geometry. Although current injectable reconstructive materials show promising defect conformity, their long-term mechanical stability remains insufficiently characterized. Current crosslinking strategies also exhibit variability in reproducibility and in vivo modulus retention. Future work must link rheological durability directly to reconstructive performance to establish more explicit design criteria.

### 2.5. Aesthetic Materials

**Figure 6:**
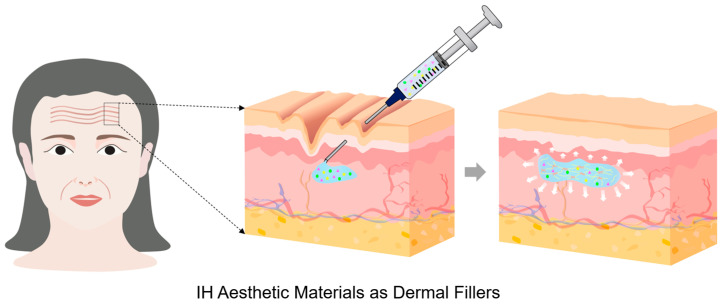
Schematic illustration of an IH-based aesthetic material, with bioactive ingredients represented by green dots, yellow five-pointed stars, and pink triangles, used as a dermal filler, administered into the dermal layer to correct wrinkles and restore soft-tissue volume for aesthetic enhancement and skin regeneration.

Conventional aesthetic materials, such as hyaluronic acid and collagen fillers, primarily act as inert volumizing agents that provide temporary correction of soft-tissue defects for cosmetic enhancement [77]. In contrast, next-generation aesthetic materials are biofunctional and regenerative systems that combine mechanical resilience with biological activity [78,79]. These systems not only restore tissue volume but also promote collagen synthesis, angiogenesis, and controlled remodeling, leading to more natural and durable aesthetic outcomes [80,81]. IHs have become central to modern aesthetic material systems, offering minimally invasive alternatives to conventional implants for soft-tissue augmentation, wrinkle correction, and facial contouring (Figure 6) [82]. In particular, IHs, such as dermal fillers, are used for various aesthetic purposes due to their adjustable viscoelasticity, degradability, excellent biocompatibility, and physical properties similar to soft tissue; however, they still face challenges in controlling post-injection persistence, degradation-induced modulus loss, and natural tissue integration. To address these issues, new IH fillers are continuously being developed based on systematic design strategies to adjust composition, crosslinking mechanisms, viscoelasticity, degradation behavior and rate, and mechanical properties [80,83,84,85]. This section introduces the latest trends in IHs used as aesthetic materials.

Pérez et al. [81] synthesized HA-based IHs formed via a Michael-type reaction between thiolated HA (HA-SH) and polyethylene glycol dimethacrylate (PEGDMA) or polyethylene glycol diacrylate (PEGDA), termed HA-DA IHs. The biphasic formulations, consisting of crosslinked HA microparticles dispersed in a non-reactive HA matrix, enhanced flowability and injection performance compared to conventional monophasic fillers. The HA-DA IHs with biphasic formulation demonstrated shear-thinning behavior, rapid viscoelastic recovery (*G*′ ≈ 270–470 Pa, ~80–90% recovery within 20–25 s at high strain, 2000%), and pH stability (~7.4) after autoclave sterilization, confirming clinical suitability. In vitro assays showed that fibroblast-laden fibrin constructs were non-cytotoxic, exhibited controlled enzymatic degradation, and maintained balanced regulation of collagen metabolism, with decreased COL1A1 and MMP1 expression, indicating reduced collagen turnover without fibrosis risk. IL-6 and COX-2 inhibition confirmed the anti-inflammatory potential, contributing to safer, longer-lasting dermal integration. These IHs demonstrated strong potential for aesthetic skin regeneration. Extending filler design toward naturally derived systems, Bai et al. [84] developed a long-lasting sericin/nano-hydroxyapatite (sericin/nHAP) IH through ultrasound-induced gelation without any chemical crosslinker, providing a safe and sustainable dermal filler for wrinkle reduction and soft tissue augmentation. The sericin/nHAP IH was formed rapidly (3–5 min) and exhibited a uniform porous structure (pore size: ~17 μm), 10-fold swelling ratio, potent antioxidant effect, and anti-inflammatory properties. In a nude mouse wrinkle model, the IH induced collagen synthesis, angiogenesis, and sustained tissue volume for over 8 weeks without inflammation. They showed elastic-dominant behavior and shear-thinning behavior. The maximum *η* values of the IHs with 0.5% and 0.25% concentrations were 102 Pa.s and 100 Pa.s, respectively. They showed their potential use as dermal fillers. In a different crosslinking strategy, Mei et al. [85] prepared a double-network (DN) IH composed of polyvinyl alcohol (PVA), sodium polyacrylate (PAANa), boric acid (BA), and nano-lignin (DL) through a simple one-pot synthesis in freeze–thaw cycles. The PVA/PAANA/BA/DL (PPBL_4_) IH integrated chemical and dynamic reversible crosslinking via borate ester and hydrogen bonds, creating a robust, self-healing structure. Incorporation of nano-lignin, rich in phenolic hydroxyl groups, significantly improved mechanical strength and toughness (tensile strength: 502 kPa & elongation: 630%), and water retention by introducing multiple hydrogen-bonding sites. The PPBL_4_ IH exhibited excellent self-healing with high recovery (96%) after a large strain. The IH maintained its structure after three freeze–thaw cycles, confirming superior network resilience and viscoelastic recovery, likely due to enhanced crosslinking density via hydrogen bonding interactions within the network. The IH exhibited elastic-dominant behavior and a shear-thinning flow profile, indicating smooth injectability. These characteristics are essential for aesthetic applications. Similarly, Shen et al. [86] developed a composite IH as a soft tissue filler by integrating amino-modified poly-L-lactic acid (NPLLA) microspheres, containing copper peptides, into an HA-based IH prepared via a Schiff-base reaction of hydrazide-modified hyaluronic acid (NHA) and aldehyde-modified hyaluronic acid (AMHA). Additionally, copper peptides provided controlled antioxidant release, protecting against ROS accumulation and promoting collagen regeneration through the TGF-β/Smad signaling pathway. The composite IHs demonstrated excellent self-healing (~95% recovery) and shear-thinning flow, enabling easy injection at low pressure (F_max_ < 10 N). The rapid *t_gel_* of the composite IHs decreased with increasing NPLLA concentration. They exhibited elastic-dominant behavior (*G*′~1000 Pa), adhesion strength up to 27 kPa, and compressive strength of 93.3 kPa. In vivo results confirmed improved dermal thickness, collagen I/III deposition, and minimal inflammatory response, establishing this system as a biocompatible, antioxidant, and mechanically reinforced soft-tissue filler suitable for long-term aesthetic restoration. In another filler system, You et al. [87] suggested a thermosensitive PCL–PEG–PCL triblock copolymer-based composite IH (EVTS-Gel) containing an extracellular vesicle (EV) derived from human adipose-derived stem cells (ADSCs) for effective collagen regeneration and wrinkle reduction. The EVTS-Gel exhibited sol-gel transition points comparable to the transition temperature (32.6 °C) of TS-Gel, forming a stable network structure, while maintaining injectability at room temperature. Both IHs showed that their complex viscosity (η*), *G′,* and *G*″ values are low at room temperature and increased with increasing temperature up to 37 °C, confirming strong structural formation once administered. There was no significant difference in rheological properties between EVTS-Gel and TS-Gel across the temperature range, indicating similar processability. In vitro tests with fibroblast-fibrin constructs showed good cytocompatibility, controlled degradation, and stable collagen metabolism. Reduced COL1A1 and MMP1 implied low collagen turnover without fibrosis, while IL-6 and COX-2 suppression indicated anti-inflammatory potential for safe dermal integration. Overall, the EVTS-Gel represents a promising dermal filler for collagen regeneration.

Lifting fillers typically require high *G*′ and elasticity for projection, whereas superficial fillers require low *G*′ but high *η* to ensure smooth contouring and prevent migration. Long-term filler performance further depends on maintaining *G*′ during degradation. Despite substantial clinical use, rheological parameters such as *G*′ and *η* are often reported inconsistently, making cross-product comparison difficult. Moreover, degradation-induced decreases in *G*′ are rarely quantified despite their clinical relevance to volume loss and retreatment. Establishing rheological standards is essential for optimizing dermal filler longevity and safety.

### 2.6. Functional Bioinks for 3D Printing

**Figure 7:**
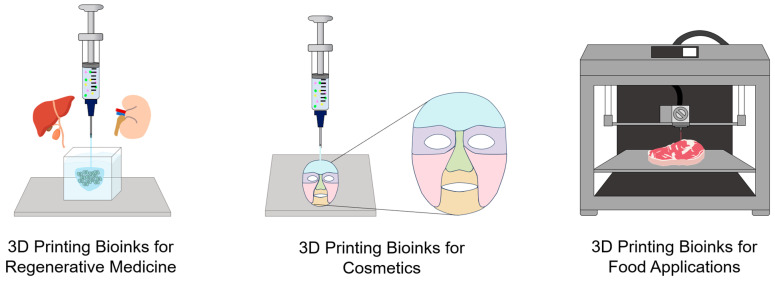
Schematic illustration of IH-based 3D-printing bioinks, with bioactive ingredients represented by green dots, yellow five-pointed stars, and pink triangles, for diverse applications, including regenerative medicine, cosmetics, and food fabrications. Three-dimensional printing IH bioinks yield precise, functional constructs, including tissue-regenerative scaffolds, customizable cosmetic masks, and structured food materials.

Recently, 3D bioprinting of IHs has attracted significant attention as an emerging biotechnology in tissue engineering, regenerative medicine, cosmetics, and food science, owing to its ability to fabricate complex, cell-loaded structures that closely mimic native biological tissues (Figure 7) [88]. This technology enables the precise fabrication of patient-specific, biomimetic tissues by printing hydrogel-based bioinks layer by layer, incorporating cells and bioactive ingredients, enabling accurate control of structure and personalized tissue adaptation [89,90]. Unlike conventional inks, which commonly face limitations in viscosity control, structural fidelity, crosslinking kinetics, and long-term biostability, potentially compromising print resolution and mechanical integrity, IH bioinks exhibit tunable viscoelasticity, cell affinity, and excellent adaptability, making them suitable for various aesthetic and regenerative applications [91,92]. Recent research focuses on optimizing the composition, rheological behavior, mechanical strength, and biological performance of IH bioinks for 3D bioprinting. Rheological properties determine printability and guiding proper crosslinking strategies. This section introduces IHs used as bioinks in tissue engineering and regenerative medicine, as well as in cosmetics and food applications.

#### 2.6.1. Bioinks in Tissue Engineering and Regenerative Medicine

IH bioinks that bridge biology and additive manufacturing provide a hydrated, three-dimensional matrix that can host viable cells and bioactive molecules. When extruded through a printing nozzle, IHs display shear-thinning flow and rapid structural recovery, ensuring precise shape fidelity [93,94]. Their ability to gel under mild, physiological conditions allows gentle encapsulation of sensitive cell types. Moreover, IHs minimize surgical invasion while conforming to irregular tissue geometries because they are injected and solidified in situ. The composition of IHs can be tuned to control mechanical stiffness, degradation kinetics, and biological signaling, enabling adaptation to diverse tissue environments [94]. Recent research has focused on integrating nanoscale fillers or bioactive components to enhance printability and tissue maturation [95]. Thus, IH-based bioinks represent a versatile and adaptive platform for tissue engineering and regenerative medicine.

Jongprasitkul et al. [93] developed a pH-responsive bioink composed of a gallic acid-functionalized hyaluronic acid (HAGA) and hyaluronic acid methacrylate (HAMA) to enhance printability and adhesion for extrusion-based 3D bioprinting. HAGA modulated pH-dependent *η*, enabling easy extrusion at 37 °C and improving tissue adhesiveness, while HAMA could be photo-crosslinked after printing and UV irradiation to form a stable network. The resulting hydrogels from the HAGA-HAMA blend bioink showed shear-thinning behavior, fast recovery (~80%), and required a low *τ*_y_ for injection, indicating smooth injectability and printability at pH 8. After UV irradiation, the hydrogels obtained from all bioink formulations exhibited high *G*′ (560–1060 Pa), elastic-dominant behavior, and faster stress-relaxation times than HAMA hydrogels without HAGA. In addition, the hydrogels exhibited strong tissue adhesion (~27 kPa) and good antioxidant properties due to the galloyl groups. The HAGA-HAMA blend bioink produced a hydrogel with tissue-adhesive and antioxidant properties, as well as tunable rheological properties suitable for 3D printing. To improve extrudability, Hull et al. [94] synthesized a dynamic IH (HELP) bioink comprising aldehyde (or benzaldehyde)-modified hyaluronic acid (HA) and hydrazine (HYD)-modified elastin-like protein (ELP). The HELP matrix was formed by crosslinking the HA and ELP via reversible hydrazone bonds. The use of modulators, competitive aldehyde analogs, and catalysts temporarily disrupted or accelerated bond exchange during printing, providing controlled gel fluidity and solidification. The reversible dynamic bonds of the HELP bioink allowed smooth extrusion under shear and post-printing recovery. In such viscoelastic crosslinked materials that exhibit self-healing behavior, modulation of bond-exchange kinetics is essential to improve extrudability and mechanical stability after printing. The bioinks showed a *G*′ of ~1000 Pa, strong shear thinning, and rapid modulus recovery (about 4 times within 24 h) following small-molecule diffusion. The optimal HELP bioink with a 50:50 ALD:BZA ratio has balanced printability and long-term stability and minimized erosion (< 3% over 14 days) while maintaining high cell viability (>95%). Cell studies using MCF10AT breast cancer cells demonstrated that the matrix enabled the spatial patterning of distinct ECM environments, where matrix viscoelasticity and integrin engagement were crucial for TGF-β–induced epithelial–mesenchymal transition (EMT). This HELP bioink showed outstanding suitability for 3D bioprinting, combining rheological tunability, mechanical resilience, and biocompatibility. In a complementary approach integrating functional fillers, Ravi et al. [95] reported a poly(ethylene glycol) diacrylate (PEGDA)-based IH bioink to mimic the critical hypoxic environment for articular cartilage tissue regeneration. The synthesized cobalt nanowires (Co NWs) were incorporated as hypoxia-inducing agents and as functional fillers to promote chondrogenic differentiation of umbilical cord-derived mesenchymal stem cells (UMSCs) that were encapsulated within the IHs. The 3D-printed hydrogel was formed by combining covalent PEGDA photo-crosslinking and physical interactions between PEG and the Co NW surface. The resulting PEGDA + Co NW IHs were transparent and porous, with a swelling ratio of ~400%, and exhibited controlled degradation over 3 weeks. The increased Co NW concentration increased the mechanical strength and structural stability of the IHs. Slow Co^2+^ release modulated cellular redox balance. The PEGDA/Co NW bioink exhibited elastic-dominant behavior and pronounced shear thinning, making it ideal for extrusion-based 3D printing. The addition of Co NWs increased *G*′ and *G*″, indicating improved network rigidity and energy dissipation capacity in the hydrogels. Moreover, the hydrogels exhibited rapid structural recovery, with the recovery ratio increasing from 2% to 98%. Consequently, the PEGDA + Co NW IH served as a hypoxia-mimicking bioink.

Ideal bioinks require strong shear-thinning behavior, rapid (<1–3 s) structural recovery, and a *G*′ that supports printed filaments without excessive deformation. Next-generation injectable hydrogels (IH fillers) combine injectability with regenerative functions, moving beyond purely volumizing materials. However, challenges persist in mechanical stability, tissue integration, and durability. Addressing these issues will require linking quantitative rheological criteria to the mechanisms of tissue remodeling. Moreover, metrics such as shear-thinning and rapid recovery are often reported without clear relevance to printing fidelity or structural stability.

#### 2.6.2. Bioinks in Cosmetics

Driven by demand for personalized skincare and improved user experience, the cosmetics industry is increasingly recognizing printable bioink-based skin-delivery systems as a key technology [96]. This approach enables the development of customized formulations that provide individualized ingredient combinations tailored to specific skin characteristics [97]. Hydrogel extrusion-based 3D printing is particularly promising, as its water-rich polymer network enhances skin affinity and enables more controlled and sustained ingredient release [98,99]. Consequently, hydrogel-based 3D printing not only enhances skin-delivery efficiency but also enables the fabrication of facial patches and masks with superior skin adhesion and personalized contours [98].

Manousi et al. [97] proposed extrusion-based 3D printed hydrogel patches designed to meet the specific needs of different skin types. The patches were formulated using iota-carrageenan (IC) as the matrix component and supplemented with sodium hyaluronate (SH), glycerol (GL), and Camellia sinensis leaf distillate (CSLD) to provide hydration, antioxidant, and sebum-regulating properties. Their structure was stabilized through ionic crosslinking with Ca^2+^ after printing, forming intermolecular hydrogen bonds between IC sulfate groups and additive hydroxyl groups. SH contributed to electrostatic interactions and hydrogen bonding with carrageenan chains, reinforcing the polymeric networks, while GL and CSLD acted as plasticizers and moisture regulators. The patches also exhibited high swelling capacity, effectively reducing sebum secretion and improving skin hydration. Three formulations were tailored, respectively, for dry (IC-SH-GL), normal (IC-SH), and oily (IC-GL-CSLD) skin, all of which exhibited excellent biocompatibility and non-irritant behavior in vivo. Rheological analysis revealed elastic-dominant behavior, thereby ensuring structural integrity during printing. The addition of SH increased *η* and stiffness due to chain entanglement, whereas GL enhanced fluidity and shear thinning, facilitating smooth printing. The optimized IC inks exhibited *η* values of 10^4^–10^5^ mPa·s, suitable for precise extrusion, and maintained a stable viscoelastic response during frequency-sweep tests. Similarly, for skin-therapeutic delivery, Graça et al. [99] developed gelatin/tannic acid (Gel/TA)-based hydrogel skin patches optimized for 3D printing, designed to treat skin irritation and inflammation such as rosacea and “maskne” caused by prolonged mask use. Gelatin provided structural integrity and biocompatibility, while tannic acid (TA) served as a natural polyphenolic crosslinker. The IH network was stabilized through hydrogen bonding and covalent linkages between TA phenolic hydroxyl groups and gelatin amine sites. In addition, metronidazole (Met) and salicylic acid (SA), as active ingredients, were incorporated for anti-inflammatory and keratolytic functions, respectively, yielding Gel/TA, Gel/TA-Met, and Gel/TA-SA hydrogel patches. Acidic groups of SAs partially disrupted hydrogel crosslinking, lowering *T_gel_*, whereas the Met enhanced hydrogen bonding, reinforcing hydrogel stiffness. All formulations exhibited elastic-dominant behavior, with pronounced shear-thinning behavior and thixotropic recovery, ensuring smooth extrusion and structural fidelity. Their gelation occurred rapidly (~25 s). This work demonstrated that the customized Gel/TA-based hydrogel patches can be effectively 3D-printed. Extending to drug-loaded dermatologic patches, Wang et al. [100] fabricated a 3D-printed niosomal hydrogel (3DP-NH) containing cryptotanshinone (CPT) for acne treatment. The niosomes were prepared using Tween 80, cholesterol, and dicetyl phosphate via reverse-phase evaporation, and the optimal system exhibited a particle size of approximately 150 nm with an encapsulation efficiency (EE) of ~67–71%. These vesicles were dispersed in a polyacrylate–glycerin hydrogel crosslinked with aluminum glycinate and tartaric acid and subsequently printed via extrusion into patch geometries. The 3DP-NH exhibited enhanced skin hydration, absence of irritation, pronounced anti-acne efficacy, and strong adhesion with excellent mechanical integrity. In vitro release followed the Korsmeyer–Peppas model, indicating Fickian diffusion with sustained CPT release (~61% over 96 h). In vivo studies on acne-induced rats confirmed enhanced anti-acne efficacy without inducing skin irritation or cytotoxicity. Rheological analysis revealed elastic-dominant viscoelastic behavior, moderate gel strength of 0.57 kg (5.59 N, 28 kPa), and stable extrudability of 0.29 kg (2.86 N) under 9–15 psi printing pressure. The formulation system exhibited shear-thinning flow, facilitating smooth printing and conformal spreading on the skin.

Optimal cell-laden bioinks maintain low shear stress during extrusion, rapid modulus recovery for shape fidelity, and a sufficient soft *G*′ to support cell survival. Hydrogel-based 3D printing enables customized skin-delivery patches with tunable viscoelasticity, print fidelity, and controlled release. However, advancing these systems requires standardized rheological criteria and improved prediction of structural and mechanical stability after printing and in vivo performance.

#### 2.6.3. Bioinks in Food Applications

Injectable and extrudable hydrogels have recently emerged as versatile materials in the food industry for applications such as 3D food printing, nutrient delivery, and texture design [101,102]. In addition to IHs, hydrocolloid-based hydrogels are particularly promising because their tunable viscoelastic properties support precise extrusion and reliable shape retention during printing [101,103]. Three-dimensional printing of food enables the digital fabrication of personalized foods with controlled shape, texture, and nutritional composition, facilitating the digitalization of food production [103,104]. Among printing methods, extrusion-based techniques are the most used, enabling rapid sol–gel transition upon deposition and ensuring structural fidelity [105]. Accordingly, IHs with proper *η* and flow behavior are considered optimal candidates for 3D food printing applications [105].

Kong et al. [102] developed pumpkin seed protein-alginate (PSP-ALG) IHs as plant-based gelatin substitutes and edible inks for 3D food printing. The stable hydrogel network was formed by dual interactions, including Ca^2+^-mediated ionic bonding and hydrogen bonding among components. The PSP-ALG IHs exhibited a gelatin-like texture, which could be tuned by varying ALG and PSP concentrations. When 2–3 wt% ALG was used, the hardness of the PSP-ALG IHs was comparable to that of gelatin (0.5–2 N). At higher ALG concentrations, the hardness and thermal stability of the IHs increased, due to protein–polysaccharide association and secondary-structure transition of PSP from α-helix to β-sheet. It was also evident that, with increasing PSP concentration, hardness decreased, attributed to the disruption of ALG-Ca^2+^ interactions. Their *t_gel_* was less than 5 min, indicating rapid network formation. The PSP-ALG IHs exhibited elastic-dominant behavior, shear thinning, and strain hardening, indicating good injectability, shape adaptability, and accuracy during 3D printing. The optimal ink (3% ALG & 4% PSP) printed stable, multilayered food scaffolds exhibiting mechanical properties comparable to those of gelatin while maintaining superior thermal resistance. This work demonstrates that the PSP-ALG IH is a suitable bioink for 3D food printing, owing to its low cost, rapid gelation, and stable network formation, highlighting its potential as a functional plant-based alternative to gelatin. Similarly, Montoya et al. [105] investigated the rheological and structural properties of starch-based hydrocolloid gel systems containing mango and arabinoxylans (AXs) to evaluate their potential for 3D food printing. The food-grade hydrocolloid gels composed of starch–mango and starch-arabinoxylan (AX) blend formulations (1–100% starch) were prepared via hydrogen bonding and chain entanglement. The gels were characterized and printed into cylindrical scaffolds under varied nozzle diameters and speeds. The hydrocolloid gels with higher starch content displayed higher *η* across the entire shear rate range, stronger shear-thinning behavior, and viscosity recovery in ~30–40 s. In addition, they showed higher *G*′ and *G*″ across the full frequency range and exhibited a denser, mesh-like microstructure with superior structural integrity compared with gels containing higher AX content. Optimal printing performance, producing more uniform constructs, was achieved with 75–100% starch blends, 0.4 mm nozzles, and printing speeds of 3–6 mm/s. As a result, thixotropy and microstructural integrity of foods were more reliable indicators of their printability than *G*′ alone, suggesting that printable food inks require a gel-like, highly thixotropic, mesh-structured network. Expanding edible bioink development, Tian et al. [106] fabricated hydrogelator inks (HIs) composed of litchi homogenate, κ-carrageenan, and xanthan gum and further developed hybrid gelator inks (HGIs) by incorporating beeswax into HIs to enhance the rheological properties and printability of litchi-based food inks for 3D food printing. The HGIs exhibited a *τ*_y_ of 623 Pa, consistency index (K) of 2.92 × 10^5^ Pa·s^n^, and *n* of 0.12, confirming strong shear-thinning behavior and enhanced structural support after printing. Compared with HIs, the HGIs showed faster *η* recovery (~30 s), a shorter *t_gel_* (~125 s), and a 10-fold higher *G*′, indicating elastic-dominant behavior. Beeswax acted as an organogelator, forming orthorhombic crystalline domains that enhanced mechanical strength, deformation resistance, and energy absorption during phase transition. Both HIs and HGIs extruded smoothly; however, HIs underwent structural collapse during successive multilayer lamination, whereas HGIs maintained their structural integrity. Printed litchi models fabricated from HGIs maintained 53.5° overhang angles and 85-layer stability without deformation, outperforming HIs in structural precision and height. Overall, the HGIs exhibiting smooth extrusion, rapid gelation, and superior self-support were considered promising material systems for precise and thermally stable 3D printing.

Food-grade IHs ideally require moderate *η* of extrusion, sufficient *G*′ for shape preservation, and controlled breakdown–recovery behavior to maintain texture. Hydrogel- and hydrocolloid-based edible inks show strong potential for precise 3D food printing by tuning viscoelasticity, shear thinning behavior, and rapid structural recovery. Printability across systems relies on forming gel-like, highly thixotropic networks that maintain structural fidelity during multilayer deposition. However, standardized rheological metrics remain limited, preventing consistent cross-study comparison and effective system optimization.

## 3. Conclusions and Challenges

Injectable hydrogels (IHs) have emerged as a highly adaptable platform that connects materials science, biomedical engineering, and aesthetic medicine. By precisely tailoring polymer composition, crosslinking mechanisms, and rheological behavior, IHs can exhibit adjustable injectability, rapid sol-gel transition, and mechanical strength and integrity suitable for various physiological environments. This review comprehensively analyzes how rheological parameters and behaviors, including viscosity, storage and loss moduli, yield stress, shear-thinning, and self-recovery, correlate with the injectability of IHs during printing or administration and with their structural integrity, degradation behavior, and biocompatibility of IHs after printing or administration. The relationship between the network structure and rheological properties of IHs was discussed across six representative application fields: hemostatic and wound dressings, tissue engineering and regenerative medicine scaffolds, controlled drug-delivery systems, reconstructive implants, aesthetic materials, and 3D-bioprinting bioinks. Across all applications, three fundamental rheological principles govern IH performance: (i) sufficient shear-thinning for low-force injectability, (ii) rapid structural recovery to ensure shape retention and mechanical integrity post-injection, and (iii) an application-specific balance of modulus, yield stress, and viscosity that determines stability, lift, diffusion resistance, and printability. These rheological characteristics determine the injectability, gelation kinetics, and long-term functional stability of IHs after printing or administration, establishing rheological characterizations as a critical link between molecular design and macroscopic performance.

Although significant progress has been made in developing IHs, particularly in their rheological behavior, the following critical challenges remain. First, achieving precise and reproducible control over viscoelastic transitions under physiological conditions is required to ensure predictable clinical performance. However, most rheological data are still obtained under idealized in vitro geometries that fail to capture confined flow, tissue micromovements, and fluid-driven changes occurring in vivo. Thus, clinically relevant factors such as pressure drop, rapid modulus, and swelling shifts upon exposure to physiological fluids must be considered in future evaluations. Second, developing self-regulating and multi-responsive IHs that adapt to complex tissue mechanics and biochemical cues is essential for advanced regenerative and aesthetic functions. Third, although microrheology provides important insight into microscale viscoelasticity, such data remain limited; thus, a related review is needed to address this issue. Fourth, from a translational perspective, regulatory compliance, batch-to-batch consistency, and sterilization-related stability strongly influence the clinical adoption of IHs by affecting network structure and rheological performance. Fifth, the establishment of standardized rheological evaluation protocols is vital for enabling cross-comparison, data integration, and the development of predictive design models.

In the future, the next generation of IHs will likely evolve from simple soft-matter systems into intelligent, mechanoresponsive, and biointeractive material systems that can sense, respond, and guide tissue regeneration or aesthetic remodeling. A more profound comprehension of the relationships among the structural architecture, rheological behavior, and functional performance of IHs, together with the integration of AI-driven material design, will continue to advance IHs toward precise tissue engineering, regenerative therapy, and aesthetic material innovations.

**Table 1:** Summary of Recent IH Systems and Their Crosslinking Mechanisms/Interactions, Key Features, and Rheological Properties for Diverse Biomedical and Aesthetic Applications.

IH Name	Crosslinking Mechanisms/ Interactions	Features	Rheological Properties	Ref.
**Hemostatic and Wound Dressings**				
N,O-CMC/ OCS	CC, SBR/	High equilibrium swelling ratio (*q*_e_ = 10–20), degradability (50–60% within 2 weeks), nontoxicity, antibacterial properties via electrostatic membrane disruption, hemostatic, wound-healing efficacy	*G*′ > *G*″ (elastic-dominant behavior), *G*′↑ & *t_gel_*↓ with OCS conc.↑, *t_gel_* ≈ 133 s (m__CMC:OCS_ = 2:1); 5 s (m__CMC:OCS_ = 1:1 or 1:2)	[39]
AC	PC/HB	Self-healing, thermo-reversible stability, antibacterial properties, hemostatic, wound-healing efficacy	*G*′ > *G*″ (*G*′/*G*″ < 10), *G*′↓ with DS↑; *G*′↑ with AC conc. ↑, *G*′↓ on heating; recovered upon cooling, *G*′ = *G*″ at *γ =* 680%, *G*′: 570→130 Pa at *γ* ≈ 1200% (*G*′ < *G*″); returns to initial at *γ* ≈ 10%, shear thinning	[42]
γ-PGA-SH/HA-CHO	CC, PC, TAAR/ HB	High water content (85–95%), antioxidant activity, tissue adhesiveness, in vivo degradation in 3 days, wound-healing efficacy, adaptability	*Tting the G*′/*G*″ > 10 in the LVE region, *G*′↓ with polymer conc.↓, *t_gel_* ≈ 3 min, lower conc. (15 → 5 wt%) → faster relaxation, shear thinning	[44]
CMC/ Eu-EDTA	PC/MCCI	Biocompatibility, self-healing behavior, real-time pH monitoring (pH 4.5–7.5), ROS generation promoting angiogenesis, theragnostic (therapy + diagnosis) for wound care, diabetic wound healing	*G*′ > *G*″ (*G*′/*G*″ < 10), *G*′↑ with Eu-EDTA conc.↑ (81.3 ± 2.2 → 236.7 ± 10.0 Pa), *G*′ = *G*″ at *γ* ≈ 300–1200%, *G*′ & *G*″ rapid recovery < 1 s (*γ*: 1% ↔ 1300%), structural recovery (within 10 min), shear thinning	[45]
4-arm PEG/PEI/ADH	CC, SBR, TTER/	Hemostatic sealant, on-demand dissolution via CME-triggered thiol-thioester exchange, high tissue adhesion, tunable mechanical robustness, viscoelasticity, biodegradability, emergency hemostatic sealant	*G*′ > *G*″ (*G*′/*G*″ > 10), *t_gel_*↓ with PEI or 4-arm PEG crosslinker conc.↑ & ADH conc.↓, shear thinning	[46]
CQCS@gel (gel: DB-PEG_2000)_	CC, PC, SBR/HB, PI, II	Biocompatibility, antioxidant effect, broad-spectrum antibacterial, pH-responsive drug release, tissue-adhesive, hemostatic, wound-healing efficacy, self-healing	*G*′ > *G*″ (elastic-dominant behavior over 600 s), *G*′↑ with CQCS conc.↑, *t_gel_* < 30 s (CQCS > 1.5 wt%), *G*′: 245 Pa (healed in 30 min) → 341 Pa (healed in 3 h); recovers to 65% and 91% of initial (373 Pa)	[47]
**Tissue Engineering and Regenerative Medicine Scaffolds**				
CS/PEG-SiW	CC, PC, SBR/ EI	Excellent mechanical strength, good biodegradability (approximately 51% within 21 days), cytocompatibility, effective promotion of cartilage regeneration, excellent flexibility, self-healing behavior, cartilage tissue engineering	*G*′ > *G*″ (*G*′/*G*″ < 10), *G*′ & compressive strength↑ with SiW conc.↑ (1 → 2%), sol-gel transition time↓ via microgel recombination, shear thinning	[48]
CSMA-PEGDA-L	CC, SBR, PIC/	High compressive strength, low cytotoxicity, excellent biodegradability, excellent therapeutic efficacy, intervertebral disc repair	Highest *G*′ with optimal 2 wt% CSMA, *η*↑ with CSMA conc.↑ (mixing↓ & crosslinking↓ at 3 wt%), *t_gel_*↓ with PEGDA conc.↑ (90 s → 36 s)	[49]
Cur@PDA@GelCA	PC/PI, HB	Exhibited excellent biocompatibility, antioxidant properties, enhanced tissue adhesion, good biodegradability (approximately 80% in 8 h), retinal tissue engineering	*G*′ > *G*″, *G*′ & compressive modulus↑ with GelCA conc.↑ (enhanced stiffness); with PDA and Cur@PDA NPs, *t_gel_*↑ with GelCA conc.↑, shear thinning	[50]
Lip/Cur + Toc@LCH, Lip/Cur + Toc@HCH	PC/HB	Excellent water absorption capacity, sustained drug release properties (10–25% for three weeks), antibacterial effect, excellent wound healing, dental tissue engineering	*G*′/*G*″ ≈ 10, *G*′, *G*″ & LVE region: Lip/Cur + Toc@LCH < Lip/Cur + Toc@HCH, *η*↑ with *T*↑ (24 °C → 40 °C), shear thinning	[51]
ACCP	CC, PC, SBR/ EI	Electroconductivity (up to 8.27 × 10^−4^ S· cm^−1^), biocompatibility, biodegradability, self-healing behavior, elastic modulus comparable to native sciatic nerve (33.5–66.8 kPa), peripheral nerve regeneration	*G*′ > *G*″ (*G*′/*G*″ > 10), *G*′↑ with CP conc.↑, *G*′ = *G*″ at *γ* ≈ 377.2%, *G*′ & *G*″ rapid recovery < 1 s (*γ*: 1% ↔ 500%), shear thinning	[56]
AHA/DTP	CC, PC, SBR/HB	Rapid gelation (~50 s), self-healing behavior (~ h), tissue-matching stiffness (2–20 kPa), biocompatibility, promotes neuronal differentiation, pH-responsive degradability, spinal cord injury	*G*′ > *G*″ (*G*′/*G*″ > 10), *t_gel_* < 50 s, *G*′ & *G*″ rapid recovery < 1 s (*γ*: 1% ↔ 450%), shear thinning	[57]
HA/PA	PC/HB	Optimized HA: PA weight ratio (4.5:1), porous interconnected microchannels (~25 μm), biocompatible, tissue adherence, regenerative medicine	*G*′ > *G*″ (elastic-dominant behavior, *G*′/*G*″ < 10), *G*′ & *G*″ rapid recovery < 1 s (*γ*: 50% ↔ 3000%), *η*_0_ = 64.85 Pa·s and *t*_R_ = 31.65 s (HA:PA = 4.5:1), thixotropic behavior, shear thinning	[58]
**Drug Delivery Systems**				
HABP-ZnBCP@CH	CC, PC, CR/ EI	Excellent adaptability to defect shape, multiple ion-delivery, strong anti-inflammatory properties, strong bone-regenerative effects, excellent biodegradability, rheumatoid arthritis therapy	*G*′ > *G*″ (*G*′/*G*″ > 10), *G*′↑ with added HABP & ZnBCP, *G*′: HABP-ZnBCP@CH (6646 Pa) > CH (3164 Pa)	[62]
NSCs-cfGel (Gel: o-Dex/4-arm-PEG-NHNH_2_)	CC/HZB	Dual drug-loaded system (cetuximab, FTY720), biocompatibility, encapsulated NSCs, tunable elasticity (G′ < 7 kPa), porous microstructure, in vivo degradation (~18 days), controlled dual-drug release (~83% released within 36 h), dynamic reversibility, spinal cord injury	*G*′ > *G*″ (elastic-dominant behavior over 1500 s at 37 °C), *t_gel_* < 2 min (at 37 °C), time-dependent *η* (~600 Pa·s), fracture stress (~4200 Pa)	[63]
Gel/HMS-ROP/DEX (Gel: OHA/CMC-ADH)	CC, PC, SBR/ HB	Self-healing behavior, tissue-adhesiveness, mechanical resilience, high ROP loading capacity (≈77%), sustained drug release (>168 h), excellent injectability, biocompatibility, controlled degradation, long-acting postoperative pain management	*G*′ > *G*″, *t_gel_* < 35 s, *G*′ & *G*″ recovery < 900 s (*γ*: 1% ↔ 200%), *G*′ (74.7 ± 4.8) recovered to 12% of the initial, shear thinning	[64]
DFO-NP/HA/F127	PC, MC/	Thermosensitive gelation, biocompatibility, minimal burst release, sustained DFO release (>14 days), 4.3-fold longer half-life vs. free DFO, robust structural integrity, treatment of iron overload disorders	*G*′ > *G*″ (in *T*: 4–40 °C); *G*′ & *η*↑ with added F127, *G*′↑ to ~8.5 kPa (DFO-NP/HA/F127, at >30 °C), sol-gel transition behavior (at >30 °C), shear thinning	[65]
HA-CPP⊂CB [8]	PC/HGI	Biocompatibility, tunable porosity, reversible self-healing, localized and sustained cytokine release (CXCL13, LIGHT), promoted TLSs formation, antigen-specific T-cell activation, tumor growth inhibition, tumor immunotherapy	*G*′ > *G*″, *G*′ & *G*″: rapid recovery <1 s (*γ*: 1% ↔ 100%), *t*_R_ = ~1 min, shear thinning	[66]
Gel@Cmab/PCZ (Gel: triblock copolymer PLGA–PEG–PLGA)	PC, MC/	Biocompatibility, biodegradability, thermosensitive gelation, sustained and localized release of Cmab and PCZ (up to 70–80% for over 15 d), enhanced ADCC in CRC, increased NK-cell infiltration, colorectal cancer immunotherapy	*G*′↑ with *T*↑, *G*′ & *G*″: low & stable (at 15–30 °C), *G*′ & *G*″: sharply↑ near at 30 °C (*G*′↑ > *G*″↑), sol-gel transition (at ≈30 °C, over 2 min), shear thinning	[67]
**Reconstructive Materials**				
D-MA + HAMA (OcuPair^TM^)	CC, PIC/	High transparency, biocompatibility, flexibility, hydration level (78–88% (similar to human cornea)), adheres to wet ocular tissue, withstands intraocular pressure > 80 mmHg, traumatic corneal injury	*G*′ > *G*′′ (elastic-dominant behavior after 40 s UV irradiation), *t_gel_* = 30–45 s (under UV light), *η* ≈ 5.0 × 10^3^ Pa·s (D-MA: HAMA = 30:70, before UV light), shear thinning	[72]
LBL	PC, MS/HB	Thermosensitive gelation, self-assembly, soft-tissue-mimicking viscoelasticity, reversible self-healing behavior, prevention of water expulsion (during gelation), excellent biocompatibility, minimal inflammation, high adaptability, reconstructive body fillers	*G*′/*G*′′ < 10, *G*′↑ with LBL conc.↑ & *ϕ*_L_↑, *T*_gel_↑ with PEG length↑ & PNIPAM length ↓, sol–gel transition (at 37 °C), J-shaped stress–strain curve	[73]
IR820/Mgel (Gel: methylcellulose)	PC/	Dual-function system (tumor recurrence prevention + breast tissue reconstruction), excellent biocompatibility, cell adhesion, long-term shape retention, sustained photothermal activity, breast reconstruction	*G*′ & *G*′′↑ with added PLGA M, sol-gel transition (at 29–31 °C), *t_gel_* ≈ 95 s (pure gel) & *t_gel_* ≈ 118 s (Mgel)	[74]
eTGF-β1 SH	/HB, HI	Excellent biocompatibility, eTGF-β1 content ≈ 5.6 wt%, sustained eTGF-β1 release (>30 days), promoted fibroblast proliferation, osteoblast maturation, osteogenic markers expression, complete alveolar bone regeneration, high structural stability	*G*′ > *G*″, shear thinning	[75]
PHM/Fe^3+^	PC/MLCB, HB	High toughness: ≈1 MJ·m^−3^, fatigue resistance (retained performance after 1000 compression cycles), minimal inflammation, thinner fibrous capsule (vs. conventional implants), elasticity (similar to soft tissue), *E*_a_ ≈ 57 kJ·mol^−1^, rapid self-recovery, breast reconstruction	*G*′ > *G*″ (*G*′/*G*″ ≈ 10, 25–60 °C), *G*′↑ with angular frequency↑, shear thinning	[76]
**Aesthetic Materials**				
HA-DA	CC, MR/	Biocompatibility, non-cytotoxicity, controlled enzymatic degradation, balanced regulation of metabolism (collagen), decreased COL1A1 and MMP1 expression, pH stability (~7.4), mechanically tunable, inflammation-modulating platform, anti-inflammatory potential, dermal filler	*G*′ > *G*″ (elastic-dominant behavior, *G*′/*G*′′ > 10), 80–90% recovery within 20–25 s after *γ* = 2000% (*G*′ ≈ 270 → 470 Pa), shear thinning	[81]
Sericin/ nHAP	PC, UIG/	Rapid gelation (3–5 min), uniform porous structure (pore size ~17 μm), 10-fold swelling ratio, potent antioxidant, anti-inflammatory properties, collagen synthesis, angiogenesis, maintains tissue volume (for 8 weeks more), dermal filler	*G*′ > *G*″ (*G*′/*G*′′ < 10), *t_gel_* = 3–5 min, *η*_max_ ≈ 102 Pa (0.5% of IH) and 100 Pa (0.25% of IH), shear thinning	[84]
PPBL	CC, PC/ BEB, HB	Self-healing behavior (96%), water retention by introducing multiple hydrogen-bonding sites, improved mechanical strength and toughness (tensile strength 502 kPa, elongation 630%), smooth injectability, dermal filler	*G*′ > *G*″ (*G*′/*G*′′ > 10), *G*′↑ added BA and DL, *G*′ & *G*″ rapid recovery < 1 s (*γ*: 1% ↔ 50, 500%), shear thinning	[85]
NHO/ NPLLA(T)	CC, SBR/	Self-healing behavior (~95%), improved dermal thickness, collagen I/III deposition, minimal inflammation, biocompatibility, antioxidant effect, dermal filler	*G*′ (~1000 Pa) > *G*″, *t_gel_*↓ with NPLLA conc.↑ (115 → 84 s), self-healing behavior (~95% recovery), shear thinning	[86]
EVTS-Gel (Gel: PCL–PEG–PCL triblock copolymer)	Self-A	Thermosensitive gelation, biocompatibility, controlled degradation, stable collagen metabolism, reduced COL1A1 & MMP1 (low collagen turnover), anti-inflammatory (IL-6 and COX-2 inhibition), promoted collagen regeneration, dermal filler	η*, *G*′ & *G*″↑ with EV, sol-gel transition at ~32.6 °C, η*, *G*′ & *G*″: low at RT → increased up to 37 °C	[87]
**Functional Bioinks for 3D Printing**				
*Bioinks in Tissue Engineering and Regenerative Medicine*				
HAGA-HAMA	CC, PIC/	pH-responsive *η*, easy extrusion (at 37 °C), tissue-adhesive, antioxidant properties, photo-crosslinked after printing and UV irradiation, self-healing behavior, tissue engineering	*G*′ > *G*″, 80–90% recovery within 20–25 s after *γ* = 2000% (*G*′ ≈ 270 → 470 Pa), shear thinning	[93]
HELP	CC/HZB	Tunable gelation via competitive aldehyde analogs and catalysts, minimized erosion (<3% over 14 days), high cell viability (>95%), mechanical resilience, biocompatibility, tissue engineering	*G*′ > *G*″ (*G*′/*G*′′ > 10), *t_gel_* = 3–5 min, *η*_max_ ≈ 102 Pa (0.5% of IH) and 100 Pa (0.25% of IH), shear thinning	[94]
PEGDA/Co NW	CC, PC, PIC/ II	Porous and transparent structure (swelling ≈ 400%), controlled degradation (3 weeks), enhanced mechanical strength, slow Co^2+^ release modulated cellular redox balance, hypoxia-mimicking for chondrogenic differentiation of UMSCs, cartilage regeneration	*G*′ > *G*″ (*G*′/*G*′′ < 10), *G*′↑ added BA and DL, *G*′ & *G*″ rapid recovery < 1 s (*γ*: 1% ↔ 50, 500%), shear thinning	[95]
*Bioinks in Cosmetics*				
IC–SH–GL/IC–SH/IC–GL–CSLD	PC/II, HB, EC	Biocompatibility, non-irritation, high swelling properties, effectively reduced sebum secretion, improved skin moisture content, skincare hydrogel patch	*G*′ > *G*″ (*G*′/*G*′′ < 10), *η* ≈ 10^4^–10^5^ mPa·s, SH addition → *η*↑ & stiffness↑, GL addition → fluidity↑ & shear thinning	[97]
Gel/TA, Gel/TA–Met, Gel/TA–SA	CC, PC/HB	Anti-inflammatory, keratolytic functions, facial skin irritation, biocompatibility, customizable formulations, hydrogel patch for treating skin lesions	*G*′ > *G*″ elastic-dominant behavior), SA addition → disrupted crosslinking & *T*_gel_↓, Met addition → enhanced H-bonding & stiffness↑, *t_gel_* < 25 s, thixotropic recovery, shear thinning	[99]
3DP-NH	CC/II	Embedded CPT-loaded niosomes (~150 nm, EE 67–71%), Sustained CPT release (~61%/96 h), non-irritant, anti-acne, enhanced skin hydration, acne therapy	Flux≈11,4 ng/cm^2^/h, *G*′ > *G*″, gel strength = 0.57 kg (5.59 N, 28 kPa), stable extrudability = 0.29 kg (2.86 N) under 9–15 psi, shear thinning	[100]
*Bioinks in Food Applications*				
PSP–ALG	PC/HB, WB	Plant-based gelatin substitute, edible 3D-printing ink, gelatin-like texture (tuned by ALG & PSP), low cost, thermally stable system, 3D food printing (pork belly, arctic surfclams)	*G*′ > *G*″ (*G*′/*G*′′ ≈ 10), PSP-ALG hardness (at 2–3 wt% ALG) ≈ gelatin (0.5–2 N), thermal stability & hardness↑ with ALG conc.↑, hardness↓ with PSP conc.↑ (ALG-Ca^2+^ disruption), *t_gel_* < 5 min, shear thinning	[102]
Starch–mango/Starch–AX	PC/PE	Food-grade hydrocolloid inks, Tunable composition, optimal printing: 75–100% starch, nozzle 0.4 mm, speed 3–6 mm/s, mesh-structured network, gel-like, high thixotropy	*G*′/*G*′′ < 10, Starch content↑ → *G*′ & *G*″↑ (stronger shear thinning; *η*: rapid recovery (*γ*: 0–200-0 s^−1^) in ~30–40 s	[105]
HGIs (HGIs: litchi homogenate, κ-carrageenan, xanthan gum, bees wax)	/HB, HI	Food-grade hybrid gelator inks, smooth extrusion, high-precision & thermally stable 3D printing, overhang 53.5° angle, 85-layer stability (HGIs > HIs), enhanced mechanical strength, deformation resistance, superior self-support	*t_gel_* < ~125 s (HGIs), *τ_y_* = 623 Pa, *K* = 2.92 × 10^5^ Pa·s^n^, *n* = 0.12, viscosity recovery (~30 s, HGIs), *G*′ (HGIs) ≈ 10 times higher than HIs, shear thinning	[106]

Abbreviations: 3DP-NH: 3D-printed niosomal hydrogel, 4-arm PEG: 4-arm poly(ethylene glycol), 4-arm-PEG-NHNH_2_: hydrazide-functionalized four-arm polyethylene glycol, AC: adenine-modified chitosan, ACCP: ALHA/CMCS/CP, ADCC: antibody-dependent cellular cytotoxicity, ADH: adipic dihydrazide, AHA: aldehyde-modified hyaluronic acid, ALG: alginate, ALHA: aldehyde-based hyaluronic acid, AMHA: aldehyde-modified hyaluronic acid, AX: arabinoxylan, BA: boric acid, CB [8]: cucurbit [8] uril, CC: chemical crosslinking, cf: cetuximab and FTY720, CH: Col-Nb/HA-Tz, Cmab: cetuximab, CMC: carboxymethyl cellulose, CMC-ADH: aldehyde-modified carboxymethyl cellulose, CMCS: carboxymethyl chitosan, CME: L-cysteine methyl ester, Co NW: cobalt nanowires, COL1A1: type I collagen, Col-Nb: collagen-norbornene, conc.: concentration, COX-2 cyclooxygenase-2, CP: CMCS-grafted polyaniline, CPP: cationic cell-penetrating peptides, CPT: cryptotanshinone, CQCS: catechol-modified quaternized chitosan, CR: click reaction, CRC: colorectal cancer, CS: chitosan, CSLD: camellia sinensis leaf distillate, CSMA: methacrylate chitosan, Cur: curcumin, CXCL13: chemokine (C-X-C motif) ligand 13, DB-PEG2000: dibenzaldehyde-terminated poly(ethylene glycol), DFO: deferoxamine, DL: dealkaline lignin, D-MA: methacrylate-functionalized hydroxyl polyamidoamine dendrimer, DS: degree of adenine substitution, DTP: 3,3′-dithiobis(propionyl hydrazide), *E*_a_: apparent activation energy, EC: electrostatic coupling, EE: encapsulation efficiency, EI: electrostatic interactions, ELP: hydrazine-modified elastin-like protein, eTGF-β1: engineered transforming growth factor β1 variant, eTGF-β1 SH: eTGF-β1 functionalized silk sericin hydrogel, Eu-EDTA: europium-ethylenediaminetetraacetic acid complex, EV: extracellular vesicle, EVTS-Gel: extracellular vesicle-bearing thermosensitive hydrogel, ƒ: frequency, F127: Pluronic F127, *G*′: storage modulus, *G*″: loss modulus, Gel: gelatin, GelCA: cinnamic acid-grafted gelatin, GL: glycerol, HA: hyaluronic acid, HABP: bisphosphonate-functionalized hyaluronic acid macromers, HA-CHO: oxidized hyaluronic acid, HA-DA: HA-SH/PEGDA, HAGA: gallic acid-functionalized hyaluronic acid, HAMA: methacrylated hyaluronic acid, HA-SH: thiolated HA, HA-Tz: hyaluronic acid-tetrazine, HB: hydrogen bonding, HCH: high-molecular-weight chitosan, HELP: aldehyde(or benzaldehyde)-modified HA-ELP, HEMA: 2-hydroxyethyl methacrylate, HGIs: hybrid gelator inks, HI: hydrophobic interaction, HIs: hydrogelator inks, HMS: hydrogel (core–shell) microsphere, HZB: hydrazone bonding, IC: Iota-carrageenan, II: ionic interaction, IL-6: interleukin-6, IR820: indocyanine green, *K*: consistency index LBL: linear-bottlebrush-linear, LCH: low-molecular-weight chitosan, LIGHT: TNF superfamily ligand, Lip/Cur + Toc: Cur and Toc-loaded liposomes, LVE: linear viscoelastic region, M: microspheres, m__CMC:OCS_: mass ratio of CMC to OCS, MA: maleic acid, MC: micellar crosslinking, MCCI: metal-carboxylate coordination interaction, Met: metronidazole, MLCB: metal-ligand coordination bond, MMP1: matrix metalloproteinase-1, MR: Michael reaction, MS: microphase separation, *n*: shear thinning index or flow index, N,O-CMC: N,O-carboxymethyl chitosan, NHA: hydrazide-modified hyaluronic acid, nHAP: nano-hydroxyapatite, NHO: NHA/OHA, NP: nanoparticle, NPLLA: amino-modified poly-L-lactic acid microspheres, NPLLA(T): antioxidant copper peptide loaded NPLLA, NSCs: neural stem cells, OCS: oxidized chondroitin sulfate, o-Dex: aldehyde-modified oxidized dextran, OHA: oxidized hyaluronic acid, PA; phytic acid, PAANa: polyvinyl alcohol, PC: physical crosslinking, PCL: poly(ε-caprolactone), PCZ: prochlorperazine, PDA: polydopamine, PE: physical (chain) entanglement, PEG: poly(ethylene glycol), PEGDA: poly(ethylene glycol) diacrylate, PEGDA-L: aldehyde polyethylene glycol (UV light), PEI: poly(ethylene imine), PHM/Fe^3+^: poly(HEMA-MA)/Fe^3+^, PI: π-π interaction, PIC: photo-initiated crosslinking, PLGA: poly(lactic-co-glycolic acid), PNIPAM: poly(N-isopropylacrylamide), PPBL: PAANa/PLA/BA/DL, PSP: pumpkin seed protein, PVA: poly(vinyl alcohol), ROP: ropivacaine, ROS: reactive oxygen species, RT: room temperature, SA: salicylic acid, SBR: Schiff-base reaction, Self-A, Self-Assembly, Sericin/nHAP: sericin/nano-hydroxyapatite, SH: sodium hyaluronate, SiW: silicotungstic acid, *T*: temperature, TA: tannic acid, TAAR: thiol-aldehyde addition reaction, *T_gel_*: gelation temperature, *t_gel_*: gelation time, TLSs: tertiary lymphoid structures, Toc: α-tocopherol, *t*_R_: relaxation time, TTER: thiol-thioester exchange reaction, UIG: ultrasound-induced gelation, UMSCs: umbilical cord-derived mesenchymal stem cells, UV: ultraviolet, UVP: UV photo-crosslinking, WB: water bridge, ZnBCP: zinc-doped biphasic calcium phosphate, *γ*: shear strain, γ-PGA-SH: thiol-modified poly(γ-glutamic acid), *η*: viscosity, η*: complex viscosity, *η*_0_: zero-shear viscosity, *η*_MAX_: maximum viscosity, *τ*_y_: yield stress, *ϕ*_L_: linear volume fraction, ↑: increase, ↓: decrease.

**Table 2:** Quantitative Rheological Properties of Natural and Synthetic IHs.

Category	Polymer Type	Crosslinking Mechanism	*G*′ [Pa]	*τ_y_* [Pa]	*t_gel_* [s]	Injectability Notes
Natural IHs	HA, Chitosan, Gelatin, Collagen, Silk, HAMA, GelMa, etc.	SBR, II, UV, HB	50–500	10–60	5–180	Excellent biocompatibility, moderate mechanical strength
Synthetic IHs	PEGDA, PVA, PNIPAM, PEG, PEI, etc.	CC, MR, UV, HB, HI	300–3000	50–200	<10–60	Tunable strength, controllable degradation

Abbreviations: CC: chemical crosslinking, GelMa: gelatin methacryloyl, HA: hyaluronic acid, HAMA: methacrylated hyaluronic acid, HB: hydrogen bonding, HI: hydrophobic interaction, II: ionic interaction, MR: Michael reaction, PEGDA: poly(ethylene glycol) diacrylate, PVA: poly(vinyl alcohol), PNIPAM: poly(N-isopropylacrylamide), PEG: poly(ethylene glycol), PEI: poly(ethylene imine), SBR: Schiff-base reaction.

**Table 3:** Rheological and Mechanical Properties Induced by Three Different Crosslinking Mechanisms of IHs.

Crosslinking Type	Crosslinking Mechanism	*t_gel_* [s]	G’ [Pa]	*Pros*	*Cons*
Physical Crosslinking	HB, II, HI	Fast (<10)	Low-mid’ (50–300)	Self-healing, Easily controllable	Weak Strength
Chemical Crosslinking	CC, SBR, MR	Slow–mid (20–180)	High (500–3000)	Strong network, High mechanical integrity	Irreversible (Exceptions exist)
Hybrid Crosslinking	Physical & Chemical Crosslinking (CC, II, HB, HI, MR, SBR)	Tunable	Tunable Range	Best Balance	Complex Synthesis

Abbreviations: CC: chemical crosslinking, *G*′: storage modulus, HB: hydrogen bonding, HI: hydrophobic interaction, II: ionic interaction, MR: Michael reaction, SBR: Schiff-base reaction, *t_gel_*: gelation time.

**Table 4:** Quantitative Rheological and Characteristic Criteria for Major IH Applications.

Application	Needle /Cannula Guage [G]	*t_gel_* [s]	*G*′ [Pa]	*F_c_* [N]	*τ_y_* [Pa]	*η* [Pa·s] at high $\overset{\cdot }{\gamma }$ = 10^2^ s^−1^	Shear Thinning	*t_R_* [s]	Note
Hemostatic Systems	20–25 [47] 20	3–205 [39] 5–133 [46] 5.3–205 [47] 18	0–10,000 [39] 0–50 [46] 10,000 [47] 100–1000	5–15	10–50	Low- Moderate (1–10)	Strong	1–5	Rapid gelation, bleeding control
Wound Dressings	22–27 [44] 22	10–60 [44] < 180	10–10,000 [42] 10–900 [44] 10–10,000 [45] 81–236	5–20	5–40	Low- Moderate (0.2–10) [44] 0.2–3 [45] 2–4	Moderate- strong [42] 5000→0.4 [44] 103→0.2 [45] 3000→2	3–10	Soft, spreadable adhesive gels
Tissue Engineering/Regenerative Scaffolds	18–23 [56] 23	20–370 [49] 36–90 [50] 28–370 [57] 30–50	30–20,000 [48] 2493–6063 [49] 35–300 [50] 291–451 [56] 122–327 [57] 2000–20,000 [58] 30	10–35	20–500 [58] 500	Low- Moderate (0.1–50) [48] 0.1–10 [50] 0.12–0.48 [51] 1–20 [57] 0.4 [58] 0.1–0.7	Moderate- strong [48] 10^5^→0.1 [50] 400→0.12 [51] 10^5^→1 [57] 1000→0.4 [58] 40→0.1	5–20	Mechanical stability for structural support
DDS	23–30 [64] 27	10–200 [63] < 120 [64] < 35 [67] < 120	100–8500 [62] 3164–6646 [63] 7000 [64] 1500 [65] <8500 [66] 1500	5–25	5–80	Tunable (1–200) [64] 20–40 (at 10 s^−1^) [66] 4	Moderate [64] 15,000→20 [66] 500→4	10–900 [64] <900	Complex Synthesis, Diffusion-dominated release
Reconstructive Materials	20–27 [73] 20–27	10–150 [72] 30–45 [74] 118	200–35,000 [72] 35,000 [73] 10–1000 [76] 10,000–30,000	10–30	10–80	0.1–150 [72] 0.7 [75] 0.02 (at 10^3^ s^−1^)	Moderate [72] 5.2→0.7 [75] 2→0.02	3–15	Mechanical strength for structural Support, shape for defect, moderate stiffness to prevent tissue necrosis
Aesthetic Dermal Fillers (Fine line)	28–32 [87] 31	5–120	50–200	8–20	5–20	Moderate -high (10–50)	Moderate	2–5	Smooth spreading and natural contouring, controllable degradation for retouch procedures
Aesthetic Dermal Fillers (Deep line)	22–27 [86] 25	5–150 [86] 84–134	100–40,000 [81] 100–400 [85] 1000 [86] 600–1500 [87] 30,000–40,000	0–35 [86] 0–10 [87] 1.2–1.4	20–120	Moderate (0.2–200+) [81] 0.45 [84] 0.2 [85] 0.09–0.38 (at 10^3^ s^−1^)	Moderate -strong [81] 40,000→0.45 [84] 200→0.2 [85] 10,000→0.09	2–10	Mechanical strength for structural Support, shape fidelity, controllable degradation for retouch procedures
3D-Printing Bioinks	18–27 extrusion nozzle [93] 27 [94] 27 [95] 20 [99] 27 [102] 25 [105] 19–27 [106] 21	< 1–300 [99] 20 [102] < 300 [106] 120–150	10–20,000 [93] 560–1060 [94] 1000 [95] 392–1046 [97] 200–1000 [105] 10–1000 [106] 7000–20,000	20–80 [106] 71	0.1–1000 [93] 0.1–100 [94] 1000 [106] 460–623	High at low shear, Low at high shear [93] 1 [94] 1 [95] 1 [97] 0.1 (at 10^3^ s^−1^) [105] 0.02 [106] 0.2	Very strong [93] 10,000→1 [94] 5000→1 [95] 100→1 [97] 2000→0.1 [105] 42,000→0.02 [106] 200→0.2	<1–3	Immediate shapeability & recovery after extrusion, Tunable strength, controllable degradation

Abbreviations: *G*′: storage modulus, *F_c_*: critical injection force, *t_gel_*: gelation time, *t_R_*: recovery time, *τ_y_*: yield stress, *η*: viscosity, [ref.].

## Data Availability

Not applicable.
